# Air-Coupled Ultrasound Systems for Biomedical Applications: Advances in Sensors, Electronic Interfaces and Signal Processing Strategies

**DOI:** 10.3390/s26051692

**Published:** 2026-03-07

**Authors:** Filippo Laganà, Riccardo Olivieri, Elena Stuppia, Gianluca Barile, Giuseppe Ferri, Salvatore A. Pullano

**Affiliations:** 1 Laboratory of Biomedical Applications Technologies and Sensors (BATS Lab), Department of Health Sciences, Magna Græcia University, 88100 Catanzaro, Italy; filippo.lagana@unicz.it (F.L.); elena.stuppia@studenti.unicz.it (E.S.); pullano@unicz.it (S.A.P.); 2 Department of Industrial and Information Engineering and Economics, University of L’Aquila, 67100 L’Aquila, Italy; riccardo.olivieri1@graduate.univaq.it (R.O.); gianluca.barile@univaq.it (G.B.)

**Keywords:** air-coupled ultrasonic transducer, biomedical sensing, AI-assisted devices, electronic interface

## Abstract

Air-coupled ultrasound (ACU) is emerging as a fully non-contact sensing modality in biomedical applications. ACU applications can be broadly classified into two main domains: (i) contactless monitoring of physiological parameters and (ii) assistive aids, robotic perception in unstructured real-world environments, enabling tracking and geometric reconstruction. Advances in electronic materials and sensor design have enhanced ultrasonic sensor characteristics (e.g., bandwidth, directivity, and intensity). In parallel, progress in front-end electronics and signal processing, including artificial intelligence (AI)-assisted analysis, has enhanced ACU performance under low signal-to-noise (SNR) conditions. This review focuses on low-frequency ACU systems, with emphasis on sensor technologies, electronic interfaces, and system architectures that enable non-contact biomedical and robotic applications.

## 1. Introduction

Air-coupled ultrasound (ACU) technology has advanced substantially in recent years and is increasingly recognized as a promising contactless modality in medical applications, alongside optical and radio-frequency approaches [1,2,3,4]. ACU enables monitoring of physiological activity by sensing subtle body-surface vibrations and micro-displacements related to respiration and cardiac motion, supporting unobtrusive vital-sign tracking and biomechanical assessment [4,5,6,7]. It also contributes to medical and assistive robotics by enabling contactless ranging, target tracking, and geometric/anthropometric reconstruction in unstructured environments [8].

Although optical and radio-frequency methods can offer higher spatial resolution, they may be more sensitive to obstructions, lighting conditions, and privacy concerns [3,4]. Considering that airborne ultrasound sensing is bio-inspired, mammalian echolocation provides a natural paradigm for contactless ranging and sensing. Low-frequency ACU systems typically operate in the 20–100 kHz band, corresponding to millimeter-scale wavelengths, and can achieve sensing distances of up to a few meters [8,9,10]. Unlike contact ultrasound, ACU operates without coupling media, enabling measurements and interaction in scenarios where contact is impractical, undesirable, or unreliable [11]. Despite focusing on different application scenarios, biomedical and robotic sensing share similarities in sensing principles and architecture, electronic interfaces, and algorithms [12,13]. These applications share a common technological challenge: achieving adequate bandwidth, directivity, and acoustic output in air [14]. Emerging trends are also evident in multimodal sensing, where ACU is combined with optical or inertial technologies [15]. Furthermore, data-driven processing techniques, such as AI-assisted micro-movement extraction, can further improve the system’s robustness and real-world applicability [16].

Advances in ACU have been closely tied to development in sensor technologies, including piezoceramics, ferroelectric polymers and micro-electro-mechanical systems/micro-machined ultrasonic transducer (MEMS/MUT) sensors, together with dedicated front-end electronics for excitation, impedance matching, and low-noise signal acquisition [17,18,19]. Piezoceramic transducers are widely used in air-coupled applications due to their high electromechanical coupling and robustness, enabling the generation of ultrasonic waves with sufficiently acoustic pressure despite the strong acoustic-impedance mismatch with air [20,21]. In parallel, MUTs are increasingly adopted for air-coupled systems because their multichannel array architectures support advanced beamforming and acoustic imaging, which are particularly relevant for medical and assistive robotics under occlusions, variable lighting, and privacy constraints [22,23]. Finally, ferroelectric polymers such as polyvinylidene fluoride (PVDF) and its copolymers (e.g., P(VDF–TrFE)) are attracting interest for airborne operation because their acoustic impedance is closer to that of air than conventional piezoceramics, potentially improving transmission efficiency and reception sensitivity [18,24]. Recent work suggests that such ferroelectric in membrane or planar array configurations can enhance acoustic coupling in air and provide high fractional bandwidths, supporting the detection of physiological micro-movements and contactless perception tasks [24,25]. Despite progress in transducer design, air propagation remains fundamentally constrained by frequency-dependent attenuation, sensitivity to temperature and humidity, and pulse dispersion, resulting in an inherent trade-off between spatial resolution and operational range [26,27,28]. In this context, the design of dedicated electronic front ends is critical to preserve weak echo signals and maximize system sensitivity under low signal-to-noise ratio (SNR) conditions. Advances in conditioning circuits and synchronized multi-channel architectures have partially mitigated these limitations, improving scalability and robustness [29]. Finally, modern ACU systems increasingly rely on advanced signal processing and knowledge-driven algorithms to extract reliable information from highly attenuated and noisy ultrasonic signals in realistic biomedical and robotic environments [30,31].

This review will focus on ACU with emphasis on sensor technologies, front-end electronic interfaces, and system-level architecture that enable robust and scalable solutions for biomedical monitoring and medical/assistive robotics.

## 2. Air-Coupled Ultrasound Sensing

### 2.1. Generation and Reception of Low-Frequency Ultrasound

**Figure 1:**
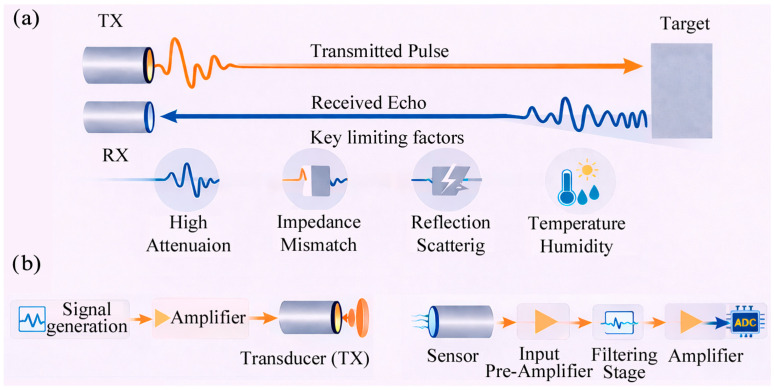
(**a**) Low-frequency ACU pulse transmission and echo reception in air, highlighting the key limitations along the signal path: High attenuation, impedance mismatch, reflection/scattering, and temperature/humidity effects. (**b**) Typical ACU architecture: Transmit chain (signal generation and power amplification driving the TX transducer) and receive chain (sensor, pre-amplification, filtering, amplification, and downstream digitization/signal processing).

Air-coupled ultrasound relies on the transmission of acoustic waves through air, a medium whose acoustic impedance is several orders of magnitude lower than that of other media (e.g., biological tissues, and most solids) [32]. This extreme mismatch is the primary physical constraint of airborne ultrasonics and leads to acoustic reflection at air–solid interfaces [17,18,19]. Ultrasonic propagation in air is strongly affected by frequency-dependent attenuation (which increases at higher frequencies), dispersion, and environmental factors such as temperature and air currents [33]. The term low-frequency ACU refers to airborne ultrasonic operation below approximately 100 kHz, with particular emphasis on the 20–80 kHz band, which represents the most adopted range for biomedical vital-sign monitoring and short-range assistive detection. Systems operating slightly above this range are also included for comparative purposes and to illustrate emerging technological trends. Figure 1a schematically shows the ACU operating principles and the main physical factors that limit pulse transmission and echo reception in air (high attenuation, impedance mismatch, scattering, and environmental effects). Although ACU has been explored across a broad frequency range, practical operation for biomedical sensing and field deployment is typically pushed toward low frequencies, often <100 kHz, because sound attenuation in air increases strongly with frequency (commonly approximated as α∝f^2^) [32,33]. A typical ACU chain starts with signal generation via pulsed, burst, or coded excitation of a transmitting (TX) element (Figure 1b). In air, the average sound speed is about 343 m·s^−1^ at 20 °C and shows a temperature dependence of roughly 0.6 m·s^−1^·°C^−1^, which must be compensated to ensure accurate pulse-echo timing estimation (e.g., time-of-flight, TOF) [34,35]. Weak echoes are converted back to electrical signals by a receiving (RX) element and conditioned through high-impedance, low-noise preamplification and band-limited filtering prior to digitization and processing (Figure 1b) [36]. Since coupling losses at the transducer–air boundary dominates airborne operation, acoustic matching layers with intermediate impedance are frequently adopted. For a single matching layer, the optimal impedance is commonly expressed as *Z*_layer_ = √(*Z*_material_ *Z*_air_) [37]. Lightweight polymeric layers are therefore attractive, and electrets (charged porous cellular polymers exhibiting piezoelectric characteristics) have emerged as effective candidates to support more efficient air coupling [38,39].

In ACU, excitation is typically tailored in terms of duration, spectral content, and modulation. In biomedical/field ACU systems, carrier frequencies are often in the 20–100 kHz range (most frequently 40 kHz) [2,10]. Constant-frequency (CF) excitation includes continuous wave tones transmitted over long intervals (tens to hundreds of ms, or longer). CF is well suited for phase/Doppler tracking (sub-mm motion) but provides limited range discrimination alone [6,40]. Short tone bursts gate the same carrier over a few to tens of cycles. Typical bursts use ~5–20 cycles, i.e., ~0.1–0.5 ms at 40 kHz. Burst length trades energy/SNR against temporal resolution and echo overlap [8,32]. Broadband transients are obtained by very short pulses or click-like waveforms. Impulse-like drives may be ~1–3 effective cycles (≈25–75 µs at 40 kHz), sharpening TOF localization [7,8]. Their effective bandwidth can reach several tens of kHz but is limited by the transducer and filtering [18,20]. Frequency-modulated (FM) signals sweep across a band (linear or non-linear chirps) [28]. Practical sweeps may span, for example, 30–90 kHz over ~1–5 ms. Such sweeps inject more energy without increasing peak pressure, thereby improving detectability in air. Matched filtering/pulse compression is used to recover a short effective echo response [10,26]. Waveform selection is a system-level choice balancing coupling losses, SNR, motion tolerance, and timing accuracy [11,21].

### 2.2. ACU Application in Physiological Monitoring and Medical Robotics

**Figure 2:**
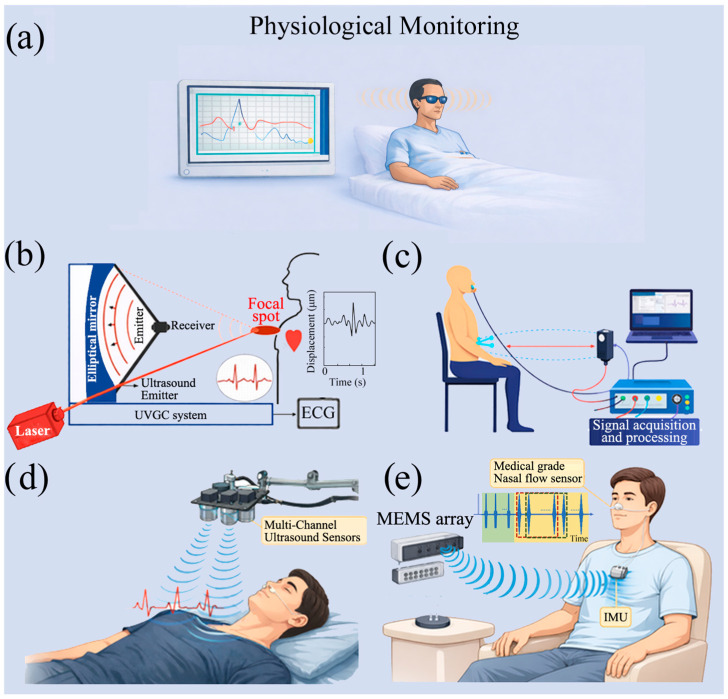
(**a**) Contactless physiological monitoring, where airborne ultrasound tracks subtle body-surface vibrations associated with respiration and cardiac activity without physical contact [4,5]. (**b**) Non-contact and through-clothing measurement of the heart rate (HR) using ultrasound vibrocardiography (adapted from [40]). (**c**) Experimental setup for non-contact respiration rate measurement system using an ultrasonic proximity sensor positioned approximately at level with the subject’s abdomen area. A nasal thermocouple sensor, affixed on the subject’s nose, is used for comparison with the US system (adapted from [46]). (**d**) System setup of a multi-channel ultrasound system for non-contact heart rate monitoring, with the transducer placed in front of the pit of the neck at a distance of about 20 cm (adapted from [42]). (**e**) An experimental setup where subject wears an IMU on the chest and a nasal flow detector while being monitored by a system for contactless respiratory waveform estimation using ultrasound planar array. The signal is a pulse sequence used in the system (adapted from [41]).

Low-frequency ACU systems are typically deployed with different operating modes depending on whether the target application is (i) contactless physiological monitoring or (ii) robotic perception in unstructured real-world environments, enabling tracking and geometric reconstruction (see Figure 2a). For contactless physiological monitoring, ACU systems are predominantly employed to track thoracic respiratory motion and, in more demanding settings, to capture heartbeat-related cardiomechanical micro-vibrations as micrometric chest-wall displacements, enabling unobtrusive vital-sign estimation without physical contact [41,42,43,44,45,46].

In physiological monitoring, ACU is primarily used to capture subtle body-surface vibrations and micro-displacements associated with respiration and, in more demanding settings, cardio-mechanical activity [4,5]. The interest in ACU systems lies in the possibility of performing prolonged, non-invasive measurements in sensitive contexts, such as home monitoring, intensive care units and situations where physical contact with the patient is critical or undesirable. In such scenarios, the robustness and stability of the system over time become as important as, if not more important than, spatial resolution.

Representative implementations commonly operate at 40 kHz and adopt a single-view ranging configuration in which a transmitter irradiates the thorax/upper-body region and a receiver collects the reflected field, enabling respiration monitoring in bed-like conditions and other unobtrusive scenarios [4,5,6,7]. In this domain, performance is rarely limited by spatial resolution; rather, the key requirements are coherent detection, high receive sensitivity, and robustness under low SNR. Accordingly, the signal processing focus is on extracting quasi-periodic motion rather than forming detailed images. Signal processing strategies typically include: (a) repeated TOF tracking to follow slow range changes over time, (b) phase and/or amplitude demodulation to enhance sensitivity to sub-millimetric motion, and (c) frequency-domain analysis (FFT or time–frequency features) to estimate respiratory rate and related descriptors from the motion signal.

Phase-based approaches have been explicitly adopted to increase sensitivity in cost-effective air-coupled breathing systems. In support of the above considerations, there have been experimental studies showing the feasibility of ACU systems in the field of non-contact physiological measurements. These studies provide concrete validation of signal acquisition and processing strategies, demonstrating that micro-vibrations and micro-displacements on the surface of the human body can be reliably detected without mechanical coupling with the subject. One of the most representative contributions is [40], where cardiac activity is estimated by analyzing the phase modulation of the reflected ultrasonic signal, which is induced by the heartbeat on the chest wall, even under the presence of clothing (see Figure 2b). The main strength of this approach is the complete absence of physical contact, making it very suited for unobtrusive long-term observation settings. However, the study also mentions the natural limitations of this method as having a high sensitivity to gross subject movement and an incomplete characterization of the electronic front-end parameters. To improve measurement robustness, a multi-channel acquisition architecture is introduced, allowing the signal-to-noise ratio to be increased through the spatial combination of Doppler signals (see Figure 2c) [41]. This strategy improves stability and reliability under low-SNR conditions, but at the cost of increased hardware complexity and power consumption, which are critical constraints for embedded and portable applications. An alternative approach, characterized by greater architectural simplicity, is proposed in [42], where heart rate estimation is based on the periodicity of Doppler frequency variations with reduced hardware. However, the strong dependence on the geometric stability of the measurement setup limits its reliability in uncontrolled contexts. A particularly significant contribution is represented by [43], which demonstrates the possibility of simultaneously extracting cardio-mechanical and respiratory information from a single ACU sensor. The major strength of this study lies in its high level of functional integration, whereas the lack of a systematic discussion on filter design and spectral separation of physiological components can be seen as a limitation in methodology. Non-contact respiratory monitoring has been extensively explored in studies such as [44,45]. In these studies, respiration is estimated by analyzing slow variations in distance or phase of the reflected ultrasonic signal. These approaches have the advantage of low cost and easy implementation but suffer from limited temporal resolution and sensitivity, with performance highly dependent on post-processing techniques. More advanced configurations, such as [46], demonstrate that the use of air-coupled ultrasonic arrays allows the entire respiratory waveform to be reconstructed, improving the temporal continuity of physiological information (see Figure 2d,e). A typical example of an ultrasound system output is shown in Figure 2b, where a displacement signal band-pass filtered between 4 and 40 Hz highlights heartbeat-related vibrations measured with the UVCG system on a standing subject. The waveform is quasi-periodic, with each burst corresponding to cardiac mechanical activity transmitted to the skin surface. As reported in [40], these displacement peaks are typically in the micrometric range (about one order of magnitude smaller than respiratory displacements), thus requiring high phase sensitivity for reliable non-contact detection. For respiration-rate measurements, Figure 2c illustrates the pulse sequence used in the proposed system, including the delta signal for time synchronization [41].

However, this improvement comes at the cost of greater hardware and computational complexity. Subsequent studies have extended the non-contact ACU techniques to enable the simultaneous analysis of multiple human body parameters, thus reducing sensitivity to operating conditions and improving overall robustness. In reference [47], a joint phase Doppler system is presented for estimating cardiac and respiratory information simultaneously, with greater stability than those that utilize different domains. Similarly, reference [48] illustrates that vital information can be obtained by combining the time of flight and phase variations. An approach specifically oriented towards Doppler-based respiratory monitoring is presented in [49], where respiration is estimated from thoracic micro-oscillations induced by the respiratory cycle. Although the architecture is simple and low-cost, performance is highly dependent on the geometry of the setup and the orientation of the sensor [50,51,52,53,54]. A comparative summary of the main non-contact ACU studies for physiological monitoring, including the stated acquisition parameters where available, is shown in Table 1.

In robotics and assistive technologies, ACU can support ranging, obstacle detection, target tracking, and coarse geometric reconstruction when optical sensing is degraded by occlusions, variable illumination, specular/transparent surfaces, or privacy constraints.

**Figure 3:**
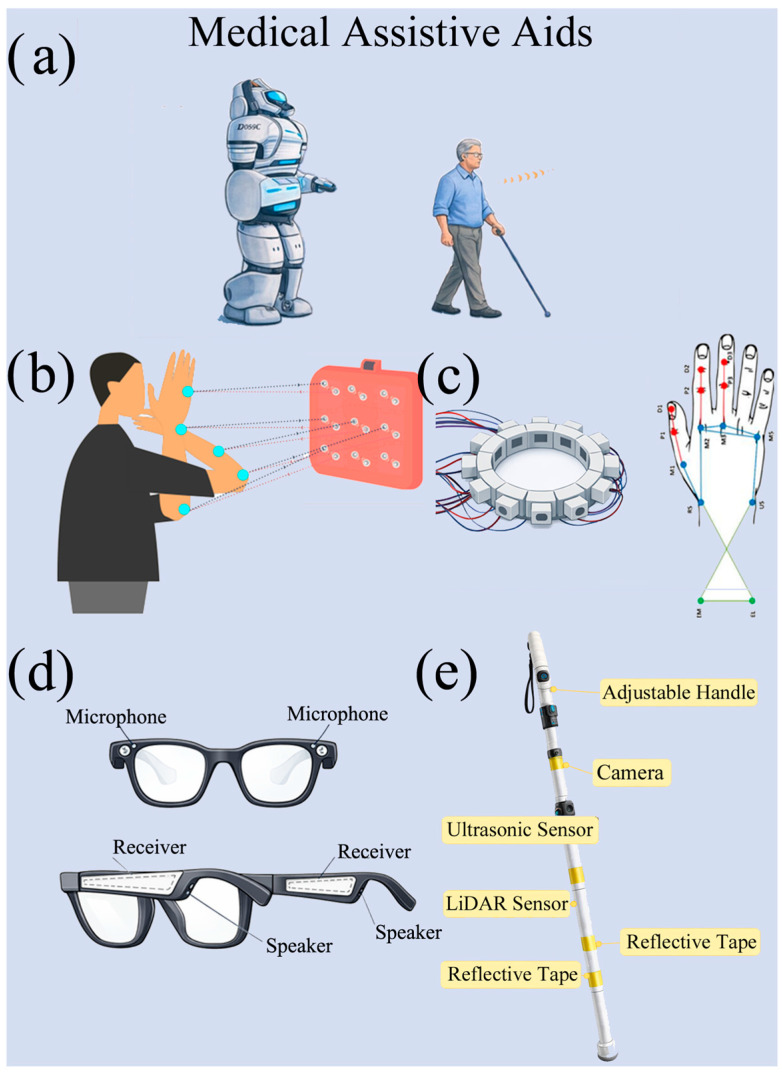
(**a**) Ultrasonic assistive aids and medical robotics, including obstacle detection and navigation in unstructured environments using biosonar-inspired time-of-flight sensing, applied to devices such as smart canes, wearable sensors, and mobile assistive aids. (**b**) Ultrasound-based gesture recognition contactless system (adapted from [55]). (**c**) Wearable ultrasound (bracelet) for wrist and hand kinematic tracking (adapted from [56]). (**d**) Smart glasses with integrated left/right microphones, receivers, and speakers adapted from [57,58]. (**e**) Smart cane with integrated sensing modules and user-feedback elements (adapted from [59,60]).

The term assistive robotics should be interpreted in a broad sense, including ultrasound-enabled environmental perception modules integrated into assistive devices and human–machine interfaces, rather than fully autonomous robotic control systems. In this environment, the emphasis shifts from maximizing sensitivity to periodic micro-motion toward reliable TOF estimation, adequate spatial coverage, and robustness to multipath and surface-dependent reflectivity (see Figure 3a). Biomimetic and sonar-inspired lines of work have demonstrated how engineered reflectors/beacons and acoustic cues can support navigation and localization performance in cluttered scenes, motivating designs that explicitly manage echo structure.

Assistive navigation devices represent a pragmatic instance of this operating mode: “smart cane” concepts leverage ultrasonic time-of-flight distance sensing to alert users to obstacles, with recent designs emphasizing usability and operation across diverse conditions. In medical and assistive robotics, similar sensing principles can be integrated on mobile platforms to provide short-range perception in patient-facing environments, where conservative sensing (e.g., presence/distance) may be preferred over imaging for simplicity and privacy.

In addition to vital-sign monitoring, ACU sensors have been used for the quantitative assessment of human movement, with relevance to neurological and rehabilitation (see Figure 3b). In [55], repetitive finger movement is quantified by analyzing variations in the flight time of the ultrasonic signal. A key advantage of this approach is its high sensitivity to micro-movements, which makes it particularly suitable for screening motor disorders. However, the limited measurement range and the need for accurate sensor alignment represent significant application constraints. Extensions to hand and gesture tracking are discussed in [26,61,62]. These studies show how the absence of wearable sensors improves user acceptability, but at the cost of lower spatial resolution compared to vision-based systems and greater sensitivity to environmental reflections. Subsequent studies have shown that the accuracy of motion detection can be further improved by analyzing micro-variations in the phase of the ultrasonic signal. In [63], the possibility of detecting micro-movements of the limbs with sub-millimeter resolution is demonstrated, making the approach particularly promising for neuromotor monitoring applications. A further extension is proposed in [59], where a hybrid strategy based on time of flight and Doppler allows dynamic tracking of fingers and hands, improving the temporal continuity of the signal compared to single-domain solutions (see Figure 3c) [55]. Beyond biomedical monitoring, ACU has also been successfully integrated into assistive technologies for users with visually impaired (see Figure 3d) [56,57]. For example, ultrasonic smart eyeglasses employ frequency modulation/demodulation to sense obstacles in both indoor and outdoor environments, providing real-time spatial cues without requiring direct contact. Another assistive application is the electronic cane equipped with ACU transducers (see Figure 3e) [57,60] which exploit TOF measurements to identify hazards ahead of the user. These devices demonstrate how ACU systems can be effectively integrated into mobility aids for enhanced environmental perception. Solutions based on air-coupled ultrasonic arrays, such as [60], show that spatial localization accuracy can be significantly improved through multi-channel acquisition and beamforming techniques. However, these benefits are accompanied by increased costs, size and power consumption, which can limit adoption in compact medical devices. An overview of representative studies dedicated to physiological and motor assessment using non-contact ACU sensors is shown in Table 2.

Studies confirm the potential of air-coupled ultrasonic sensors for non-contact biomedical sensing while also highlighting marked heterogeneity in system architectures and reporting practices. From a system perspective, these robotic operating modes more frequently benefit from synchronized multi-channel acquisition and, where available, array-based operations to improve directivity control, suppress spurious echoes, and stabilize tracking in dynamic scenes. However, current research has revealed that multi-channel arrays and beamforming techniques can significantly improve the ranging accuracy of the ACU system when operating in different environments. It is important to note the need for an integrated design of the electronics, sensors, and algorithm in achieving the highest possible accuracy for both healthcare and robotic applications [4,5,6,7,8]. In summary, ACU sensing is inherently constrained by airborne propagation physics, resulting in low signal amplitudes and environmental sensitivity. These limitations motivate a system-level optimization approach in which sensor technologies, electronic interfaces, and signal processing techniques are jointly designed to achieve robust performance in biomedical and robotic applications. Studies have shown that a biomedical application of ultrasound is echolocation to provide human users with environmental cues rich in objective and spatial information that are more elaborate than other assistive devices. The devices in question are equipped with wearable headset with an ultrasonic emitter and a microphone with an artificial earcup attached [53]. In nature, the most sophisticated echolocation abilities are found in some animals such as some species of bats, dolphins and whales. The basics of human echolocation, on the other hand, remain poorly characterized and most of the existing literature suggests that the human echolocation capacity does not come close to the precision and versatility found in highly specialized organisms. The ultrasonic pulses employed by echolocalized animals produce higher spatial resolution, stronger directionality, and higher bandwidth than human-audible frequencies. Inspired by the biological mechanism of bats and cetaceans, these systems rely on the deliberate emission of ultrasonic pulses—typically ranging from 20 kHz to 200 kHz and up to 2 MHz in case of high-resolution medical imaging—to map the environment and detect obstacles. The principle involves computing the TOF between the trigger of an ultrasonic burst and the reception of its reflected echo, a process that requires precise synchronization and specialized electronics stages [62]. Echolocation systems offer the possibility to acquire anatomical and biomechanical information from tissue without direct coupling, indicating potential for advanced biomedical localization and allowing the realization of assistive human echolocation devices.

## 3. Sensor Technologies

### 3.1. Sensor Design and Characterization

**Figure 4:**
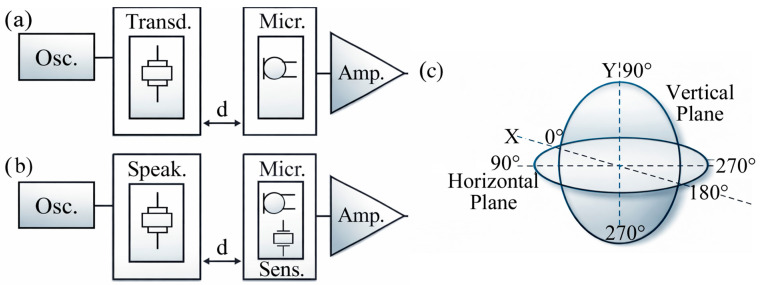
(**a**) Transmission–reception configuration used to characterize the SPL of an ultrasonic transducer, consisting of a signal generator (oscillator), a piezoelectric transducer, a reference microphone placed at a fixed distance d, and a signal amplification and acquisition stage. (**b**) Measurement configuration for sensitivity employing a loudspeaker as acoustic source and a sensor under test as receiver, with the microphone used for reference calibration at distance d. (**c**) Definition of the angular reference system and measurement planes used for directivity and sensitivity characterization, showing horizontal and vertical planes (adapted from [75]).

The characterization of air-coupled ultrasonic sensors relies on a combination of electrical, electromechanical, and acoustic parameters (e.g., resonance frequency (SPL), receiving sensitivity, and spatial radiation), which represent the fundamental metrics for comparing different sensor technologies [64,65,66,67,68,69,70]. The resonance behavior of ultrasonic sensors is commonly analyzed using equivalent circuit models, such as the Butterworth–Van Dyke (BVD) model, which describes the electromechanical coupling through a resonant branch in parallel with static branch [69,70]. Experimentally, the complex input impedance or admittance of the transducer is evaluated as a function of frequency. The resonance frequency is identified at the zero crossing of the imaginary part of the motional impedance, corresponding to the condition of purely resistive behavior [71,72]. From the frequency response, additional parameters such as motional resistance, capacitance, inductance, and quality factor can be extracted, providing insight into bandwidth and energy losses. The acoustic output of an air-coupled ultrasonic transmitter is quantified in terms of sound pressure level. SPL measurements are performed at a fixed reference distance, typically 30 cm, under free-field or anechoic conditions [73]. SPL and emitted pressure are both expressed in decibels (dB) and are relative to the standard reference pressure of 20 µPa (see Figure 4a).

Frequency-dependent SPL measurements allow the evaluation of transmission efficiency and usable bandwidth, which are critical in air due to strong frequency-dependent attenuation [74,75]. Receiving sensitivity is defined as the electrical output generated by the sensor in response to a known acoustic pressure [76]. Sensitivity is commonly expressed in Volt per Pascal (e.g., 10 V/Pa) or in decibel (see Figure 4b). Measurements are performed at the same reference distance used for SPL characterization to ensure consistency. Spatial characterization is carried out by measuring the angular dependence of the transmitted and received ultrasonic fields. Radiation patterns in transmission and sensitivity patterns in reception are typically acquired by rotating the sensor or the reference microphone over defined angular ranges on orthogonal planes (horizontal and vertical); see Figure 4c. Polar measurements yield beam-pattern diagrams that quantify beamwidth, directivity, and angular coverage, which are relevant for curved or non-uniform air-coupled ultrasonic geometries [77,78]. Since propagation in air is strongly affected by attenuation, dispersion, and environmental conditions (e.g., temperature and humidity) [79], sensor characterization is often complemented by system-level tests that assess the sensor together with the electronic interface and signal conditioning chain, providing a realistic estimate of sensitivity and signal-to-noise ratio [80].

### 3.2. Sensors Materials

ACU transducers are typically fabricated using materials with either an acoustic impedance comparable with that of transmission medium or electromechanical properties capable of reducing the mismatch between transducers element and air [21]. The material most employed in ACU transducer design includes piezoceramics, piezoelectric composites, piezopolymers, polypropylene ferroelectret foams, silicone or silicone rubber combined with semiporous membranes and aerogels [81,82].

Table 3 summarizes the main material classes reported in the literature, along with their functional roles, advantages, and limitations.

Among the most widely used materials are piezoelectric, which are sometimes combined with matching layers to better adapt the acoustic impedance between air and material, avoiding massive transmission losses [83]. Ultra-low impedance materials, such as ferroelectric foams and aerogels, have played a decisive role in mitigating the intrinsic misalignment between solid transducer elements and air, enabling broadband emission and significantly improved sensitivity compared to traditional piezoceramics. Recent studies further indicate that integrating ACU with computational imaging techniques, in particular with deep neural networks trained on spatiotemporal ultrasonic signatures, makes it possible to compensate for multipath propagation and atmospheric turbulence, significantly improving reconstruction quality in low-SNR conditions typical of the air environment [84,85].

An example of a matching layer is based on epoxy resin filled with hollow polymer microspheres [86], which has a substantially lower acoustic impedance than piezoelectric materials (0.45 MRayl). Epoxy resin is also used combined with various fillers such as aluminum powder, aluminum-oxide (Al_2_O_3_) particles or tungsten powder [87]. In case of high-temperature transducers, an organosilicon substrate is used as a single acoustic matching layer. Hollow glass microspheres (embedded in epoxy or organosilicon matrix) are used to obtain low-impedance matching [88]. To achieve a smooth transition between the high acoustic impedance of the transducer and the low impedance of the propagation medium, thus maximizing energy transfer over a wide range of frequencies, several different layers are also used together: for example, the aluminum alloy AlSi10Mg is used, which has an inherently high acoustic impedance (16.7 MRayl), and a standard epoxy resin that acts as a low impedance (2.7 MRayl) component (often used as a matrix polymer in contrast with high-impedance metal particles). The epoxy portion is dominant on the side that interfaces the air, and its percentage gradually decreases as it approaches the metal side of the alloy. This results in a continuous variation that ensures better acoustic transmission over broadband [89]. Ferroelectret materials are non-polarized dielectric polymers such as polypropylene (PP), polyethylene (PE), polyurethane (PU), Cyclo-Olefin Polymer (COP/COC), PTFE/FEP (fluoropolymers), polydimethylsiloxane (PDMS), and PVDF copolymers (with also a real ferroelectric component) [90]. They have a closed porous structure with smooth inner walls and features that make them better than classic ferroelectrics. Piezoelectricity comes from the deformation of pores, rather than from the movement of atomic dipoles as in ferroelectrics. They have a very high piezoelectric coefficient, very low dielectric permittivity, and are very light and flexible. The main advantage is the low acoustic impedance (≈0.03 MRayl), which is much closer to that of air [91]. The emergence of aerogel-based adaptation layers, whose acoustic impedance is close to that of air, has enabled near-ideal transmission characteristics, enabling broadband air sensing that was not achievable with conventional polymer interfaces [92].

### 3.3. Piezoceramic Sensor Design

Ultrasonic transducers based on piezoceramic materials, most commonly lead zirconate titanate (PZT), represent one of the earliest and most widely adopted solutions for air-coupled ultrasonic applications [88]. Owing to their high electromechanical coupling coefficients and mechanical robustness, PZT transducers can generate high acoustic pressure levels in air. However, the large acoustic impedance mismatch between PZT ceramics (≈30 MRayl) and air (≈0.0004 MRayl) requires the use of dedicated mechanical and acoustic structures to ensure efficient sound radiation. To mitigate impedance mismatch, air-coupled PZT transducers are commonly implemented using horn-loaded architectures. In these designs, a thin or bulk PZT ceramic element is bonded to a mechanical horn that acts as an impedance transformer, progressively matching the high mechanical impedance of the ceramic to the low impedance of air [93].

**Figure 5:**
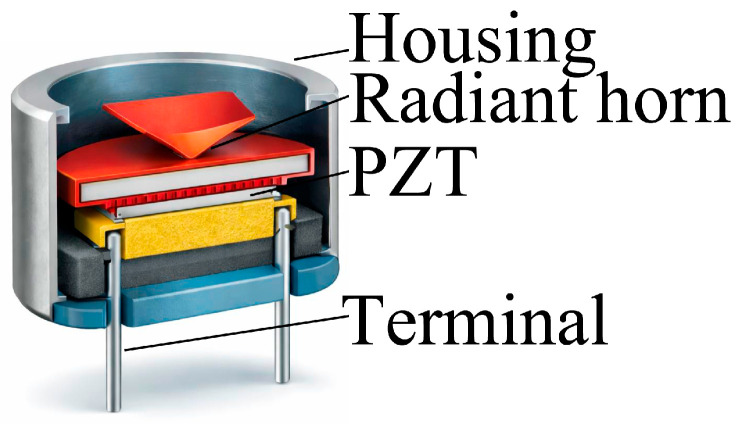
3D representation of a horn-loaded piezoceramic ultrasonic transducer for air-coupled applications.

Horn geometries are typically conical, exponential, or stepped, and are often combined with resonant cavities and dense backing materials to enhance forward radiation and suppress backward emission (see Figure 5).

The horn length is frequently designed according to a quarter-wavelength (λ/4) criterion at the target operating frequency, enabling resonant amplification of particle displacement at the horn mouth. This design criterion derives from acoustic transformer theory, according to which maximum displacement amplification occurs when the horn length is set to λ/4, ensuring constructive interference and optimal impedance transformation between the piezoelectric element and air. As a result, horn-loaded PZT transducers are usually narrowband devices operating at discrete frequencies, most commonly in the range of tens to hundreds of kilohertz. Despite their limited bandwidth and relatively large physical dimensions, these devices remain attractive for air-coupled applications requiring high acoustic output, long-range transmission, and mechanical robustness. Air-coupled horn-loaded PZT transducers typically employ a piezoelectric ceramic with a diameter in the range of 5–15 mm or 20–30 mm, and a thickness generally between 0.3 and 2 mm. The electrode materials used are commonly silver or nickel. The horn is usually fabricated in aluminum, steel or titanium and can feature conical, exponential or stepped geometry. In quarter-wavelength designs, horn length is approximately equal to λ/4 at the operating frequency, while the overall horn length typically ranges from 10 to 100 mm. The backing material consists of dense, highly lossy metals or composite materials. These transducers usually operate at frequencies between 20 and 200 kHz and are designed for resonant, narrowband operation.

### 3.4. Piezopolymer Sensor Design

**Figure 6:**
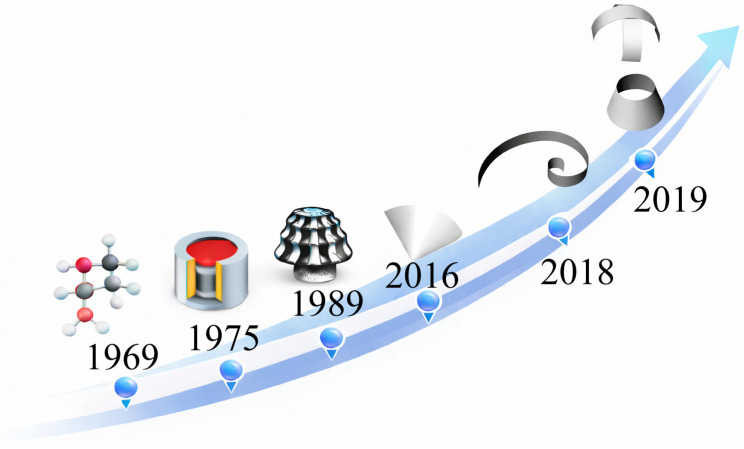
Evolution of PVDF ultrasonic transducer geometries for air-coupled applications along the timeline shown. In 1969, the discovery of PVDF piezoelectricity enabled lightweight piezopolymer ultrasound transducers. Early air-coupled implementations adopted cylindrical shells (1975), followed by hemi-cylindrical geometries (1989) to improve beam shaping and sensitivity. Semi-conical designs (2016) introduced additional control over directivity and spatial coverage. From 2018 onward, truncated-conical and spiral-shaped architectures were proposed to further tailor bandwidth and radiation patterns, culminating in quasi-spherical concepts aimed at widening angular coverage under air-propagation constraints [95].

Piezopolymer materials, particularly PVDF and its copolymers, have been extensively investigated as enabling technologies for ACU sensing due to their low acoustic impedance, mechanical flexibility, and broadband response [94]. Since the first demonstrations of curved and cylindrical PVDF film transducers in the 1970s, these materials have emerged as a viable alternative to conventional piezoceramic devices for ultrasonic operation in air, especially in the low-frequency range (20–100 kHz) (see Figure 6, Table 4) [95].

Unlike piezoceramics, PVDF-based sensors primarily exploit extensional (d_31_) vibration modes. When combined with tailored geometries—such as cylindrical, hemi-cylindrical, semi-conical, truncated-conical, spiral, and quasi-spherical structures—these modes enable efficient acoustic radiation in air while maintaining low-quality factors and wide fractional bandwidths. The evolution of sensor design has therefore been largely geometry-driven, aiming to optimize bandwidth, sensitivity, directivity, and sound pressure level through control of bending radius, clamping conditions, and effective radiating aperture.

PVDF-based air transducers mitigate the air–solid impedance mismatch through a combination of material selection and structural design. The main strategies include adding ultra-low-impedance front interfaces (e.g., ferroelectret foams, porous membranes, or aerogel-like layers) that approximate the ideal intermediate impedance between the active element and air and can be designed as quarter-/half-wavelength resonant matching structures. Additional approaches involve the use of lightweight backing/cavity structures that tune the radiation impedance and suppress backward radiation, improving forward SPL and reception sensitivity. These solutions are often co-designed with high-impedance, ultra-low-noise electronics, since PVDF receivers typically exhibit a high source impedance and predominantly capacitive behavior, making the electro-acoustic transfer particularly sensitive to parasitic and preamplifier loading [18,23,37,90,91]. More recent developments have introduced non-uniform curvature and bio-inspired geometries, including spiral-shaped and quasi-spherical transducers [96]. These configurations support multiple resonance modes, enabling broadband operation and quasi-omnidirectional radiation patterns. Such features are particularly advantageous for ACU applications characterized by strong attenuation and low SNR, including biomedical monitoring, robotic perception, and biomimetic sonar [97,98]. From a system-level perspective, piezopolymer ultrasonic sensors are especially attractive as receivers, owing to their low permittivity and high open-circuit voltage sensitivity [99]. However, their lower electromechanical coupling compared to piezoceramics makes overall performance strongly dependent on the design of high-impedance, ultra-low-noise electronic interfaces [100]. Consequently, recent works increasingly adopt co-design strategies that jointly address transducer geometry, electronic front-end optimization, and advanced signal processing to overcome the intrinsic limitations of ultrasonic propagation in air.

PVDF in-air ultrasonic transducers have been explored both for contactless monitoring of small surface motions relevant to physiological sensing [18] and for pulse–echo ranging/localization within assistive and robotic perception frameworks inspired by biosonar [97,98]. Geometry-tailored architectures—such as truncated-conical and spiral transducers—further demonstrate how curvature can be engineered to satisfy application-driven requirements on beam pattern, coverage, and sensitivity [75,96]. Although several PVDF-based copolymers have been reported to exhibit enhanced piezoelectric properties compared to pristine PVDF, their large-scale availability and commercial adoption remain limited, which has so far constrained their widespread use in practical air-coupled ultrasonic transducer implementations.

### 3.5. MEMS-Based Sensor Design

MEMS ultrasonic transducers have emerged as a complementary technology to piezopolymer-based sensors for ACU. In the context of biomedical ACU, the low frequency range is typically in the range 20–100 kHz, where air attenuation is manageable for clinical distances. While traditionally dominated by piezopolymers, MEMS ultrasonic transducers have emerged as a complementary technology to illustrate scaling trends and integration capabilities particularly in applications requiring miniaturization, dense arrays, and electronic beamforming [101,102]. Although some micromachined architectures are designed for high-frequency operation, they are included here as comparative benchmarks for sensitivity and fabrication versatility at the lower end of the ultrasonic spectrum. The two main architectures—capacitive micromachined ultrasonic transducers (CMUTs) and piezoelectric micromachined ultrasonic transducers (PMUTs)—differ in transduction principle, material stack, and achievable performance in air [103]. To maintain consistency with typical ACU scenarios, we focus on designs optimized for sub-MHz operation. CMUTs typically rely on electrostatically actuated micromachined membranes and are often operated in off-resonance conditions to achieve wide fractional bandwidth. Representative air-coupled CMUT designs report membrane lateral dimensions on the order of 32 × 32 µm^2^ with vacuum gaps around 250 nm, enabling broadband operation. As an example, an electrostatic air ultrasonic transducer with an active area of 3.3 × 3.3 mm^2^ operating at 40 kHz achieved approximately 82 dB SPL (re 20 µPa) at 8.9 cm under a 24 V bias. PMUTs exploit thin-film piezoelectric stacks deposited on micromachined membranes and typically operate in flexural vibration modes. Compared to CMUTs, PMUTs generally require lower driving voltages and simpler electronic interfaces [71]. From an acoustic matching perspective, MEMS transducers rely on microscale compliance and packaging-level design to improve coupling to air. In air-coupled CMUTs, a low-mass membrane suspended over a vacuum/air gap exhibits a low effective mechanical impedance; when operating near resonance (or in broadband off-resonance regimes), the membrane velocity can be maximized, increasing the radiated particle velocity despite the residual impedance discontinuity at the air interface [103,104]. PMUTs achieve a similar effect through flexural-mode diaphragms, typically requiring lower drive voltages while enabling large surface displacements in the low-ultrasonic band. In both cases, array architectures are a key “system-level matching” tool: a larger effective aperture and electronic beamforming increase directional gain and SNR, partially compensating for the intrinsic transmission loss at air–solid boundaries [22,103,104]. Finally, in practical MEMS modules, the acoustic window/encapsulation stack often constitutes the dominant matching interface. Low-impedance polymers and thickness tuning (often around a quarter-wavelength condition) are used to reduce front-surface reflections, while baffles, backing cavities, and isolation trenches help to mitigate substrate-borne leakage and inter-element coupling that would otherwise degrade directivity and sensitivity [105].

Air-coupled PMUTs based on single-crystal PZT have demonstrated sound pressure levels up to 100.3 dB SPL at 40 kHz measured at 33 cm, while ScAlN thin-film PMUT arrays have reported SPL values exceeding 120 dB at 10 cm, highlighting the benefits of array-based actuation at low ultrasonic frequencies (see Table 5) [104]. By contrast, PVDF piezopolymer transducers exploit geometry-driven resonance using thin poled films operating primarily in d31 mode. Typical constructions reported in the literature include 40 µm PVDF films with ~200 nm aluminum electrodes for hemi-cylindrical devices, 28 µm films for semi-conical geometries, and 30 µm films with ~5 µm silver-ink electrodes for truncated-conical designs, with operational frequencies spanning approximately 20–100 kHz depending on curvature and boundary conditions. MEMS technologies, typically associated with high-frequency imaging, can be adapted to operate in the 20–100 kHz range of curved PVDF sensors, enabling more integrated bio-acoustic platforms. Array crosstalk can be substantially reduced by using AC-PMUT cells with coaxial annular and circular diaphragms driven in anti-phase, which suppresses mutual-radiation impedance and improves SNR [105]. Crosstalk can be further mitigated by increasing inter-cell spacing (low-density arrays) and by introducing isolation trenches at diaphragm edges to attenuate edge-wave coupling [105]. Finally, both CMUT and PMUT membranes exhibit inherent nonlinear dynamics (amplitude-dependent resonance shifts), which can be modeled with Duffing-type behavior and should be considered in precision beamforming and drive-signal design [106].

### 3.6. Acoustic Matching Strategies and Comparative Advantages

To complement the horn-based impedance transformation described for piezoceramic sensors (Section 3.3), this section reviews the main acoustic-matching methodologies adopted by piezopolymer and MEMS air-coupled ultrasonic transducers and provides a concise comparison of their main technical advantages in airborne operation [18,23,93,95,103,104]. The comparison of Table 6 highlights that the “matching lever” shifts from primarily mechanical impedance transformation in horn-loaded PZT devices to material/geometry-driven compliance in PVDF films and to membrane/packaging co-design plus array gain in MEMS transducers. Consequently, horn-loaded PZT remains preferred when maximum SPL and range are required, PVDF solutions are attractive for broadband and lightweight receivers, and MEMS arrays offer the most promising route toward compact multi-channel systems with adaptive directivity control and beamforming capabilities [18,95,103,104].

## 4. Electronic Interface for Ultrasonic Sensors

### 4.1. Electronic Front Ends

Low-frequency ACU sensing represents one of the most challenging operational regimes for ultrasonic systems from an electronic interface perspective. Unlike contact-based ultrasonic techniques, where acoustic coupling media such as water or gels enable relatively efficient energy transfer, ACU systems must operate across a severe acoustic impedance mismatch between air and solid media. This mismatch results in extremely high transmission losses, which may easily exceed 80–100 dB depending on frequency, propagation distance, and target properties [107]. Consequently, the amplitude of the acoustic wave reaching the receiving transducer is reduced, and only a small fraction of the transmitted energy is converted back into an electrical signal.

From an electronic perspective, this attenuation fundamentally changes the role of the receive chain. Since the electrical signal generated at the transducer terminals is often close to the intrinsic noise floor of conventional analog front-end circuits, signal extraction is highly dependent on the electronic interface characteristics. Accordingly, the front-end cannot be regarded as a signal-conditioning stage of secondary importance; instead, they become a primary performance-limiting element of the entire sensing system. Furthermore, marginal improvements in front-end noise performance or impedance matching can provide significant gains at the system level, in some cases comparable to those obtained through direct transducer optimization.

**Figure 7:**
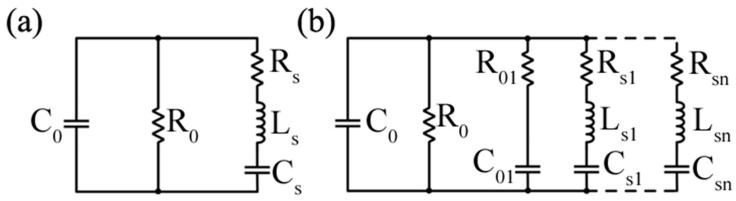
(**a**) Butterworth-Van Dyke model for hemi-cylindrical PVDF transducer. (**b**) Butterworth-Van Dyke model for spiral-shaped PVDF transducer.

These challenges are further compounded by the electrical characteristics of transducers commonly employed in low-frequency ACU applications. Piezoelectric polymer sensors, such as PVDF, are widely used because of their broadband response, mechanical flexibility, and more favorable acoustic impedance relative to air. However, from an electrical point of view, these devices typically exhibit high output impedance and predominantly capacitive behavior. When interfaced with non-ideal electronics, this combination can lead to drawbacks such as signal loading, bandwidth reduction, and increased susceptibility to parasitic capacitances associated with cables, packaging, and input devices. It is therefore essential to develop an accurate circuital-level equivalent model of the sensor. This is especially complex since it not only depends on the material used, but also on the actual shape of the transducer, as shown in Figure 7 where an equivalent circuit is shown for PVDF-based hemi-cylindrical (Figure 7a) and spiral (Figure 7b) shapes.

Noise considerations are particularly critical in low-frequency ACU systems as well. In many practical scenarios, electronic noise dominates over acoustic noise sources, especially at low frequencies where environmental and mechanical disturbances may also contribute. Analytical and experimental studies indicate that the total noise floor results from a complex interaction between the thermal noise associated with the real part of the transducer impedance, the voltage and current noise of the active devices, and additional contributions introduced by biasing networks and feedback components [107].

Nevertheless, a generic receiving chain for ultrasonic sensing comprises the front-end preamplifier, typically a very low-noise, relatively low-gain stage, intermediate analog conditioning stages, and the digitization and post-processing blocks (see Figure 1b in Section 2.1).

While this architecture is common to most ultrasonic systems, its implementation in low-frequency ACU applications is subject to significantly tighter constraints. In particular, the first stage plays the most important role, as it directly interfaces with the transducer and largely determines the effective sensitivity, bandwidth, and noise performance of the entire system. The primary role of the front-end preamplifier is to convert the weak electrical signal generated by the ultrasonic transducer into a form suitable for further processing, while preserving as much signal integrity as possible. In ACU systems, this task is complicated by the combination of very low signal amplitudes and high source impedance. Consequently, the preamplifier must provide sufficient gain without excessively loading the transducer, while simultaneously maintaining a low input-referred noise across the frequency band of interest.

**Figure 8:**
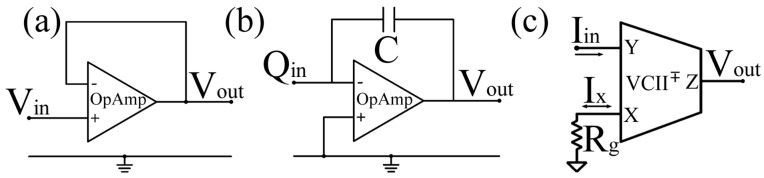
(**a**) Voltage buffer configuration ensuring high input impedance and low-noise characteristics. (**b**) Op-Amp-based charge amplifier configuration. (**c**) VCII-based transimpedance configuration.

Recent reviews on ultrasonic preamplifier design emphasize that the optimal receive chain architecture is highly dependent on the electrical nature of the transducer and the target application [108]. The choice of topology often depends on the transducer type; for instance, voltage amplifiers (VA) are typically preferred for piezoelectric sensors like PMUTs, while charge-sensitive amplifiers (CSA) or transimpedance amplifiers (TIA) are better suited for CMUT that generate currents [107]. Voltage-mode front ends rely on high input impedance amplifiers to sense the voltage generated by the piezoelectric transducer while minimizing signal loading. In principle, this approach is well suited for piezoelectric devices, which can be modeled as voltage sources in series with a capacitive kind of impedance. In practice, however, the extremely weak signals encountered in ACU systems require high gain already at the first amplification stage. Owing to the finite gain–bandwidth of operational amplifiers, increasing the closed-loop gain directly reduces the available bandwidth, thereby limiting the ability to process broadband ultrasonic signals. Figure 8a shows a basic example circuit for the voltage mode approach: a voltage buffer that ensures an extremely high input impedance while maintaining very low-noise characteristics, thanks to its unitary gain.

Charge-sensitive amplifiers have been widely proposed as an alternative to mitigate these limitations. By integrating the charge generated by the transducer into a feedback capacitor, CSAs offer reduced sensitivity to input capacitance variations and improved robustness against cable and parasitic capacitances. Comparative studies have shown that, under the presence of large parasitic capacitances, charge amplifiers can outperform simple voltage amplifiers in terms of stability and noise behavior [109]. Despite these advantages, charge-based architecture does not eliminate the fundamental limitations imposed by voltage-mode operation. The achievable bandwidth of a CSA remains constrained by the open-loop characteristics of the operational amplifier, and the feedback network introduces additional noise sources that may become dominant at low frequencies. Figure 8b shows a basic example circuit for the “charge mode” approach, where charge is evaluated via an Op-Amp-based charge amplifier.

The discussed limitations of conventional voltage-mode and charge-based front-end architectures have motivated an increasing interest in alternative interface architectures. Among these, current-mode and mixed-mode read-out systems have emerged as particularly attractive solutions for ultrasonic sensing, especially under the extreme operating conditions imposed by low-frequency air-coupled applications. Current-mode front ends differ from traditional voltage-mode approaches because the signal generated by the transducer is processed primarily in the current domain rather than being immediately converted into a voltage through high-impedance amplification. This shift offers several intrinsic advantages. First, current-mode architectures are not constrained by gain–bandwidth product limitations, as signal amplification is not strictly tied to high loop gain and global feedback. As a result, wideband operation can be achieved without sacrificing gain, a property that is particularly beneficial in broadband ACU systems where both sensitivity and temporal resolution are critical. From a noise point of view, current-mode processing can interact with high-impedance and capacitive transducers. By presenting a low-impedance input node to the sensor, current-mode interfaces can reduce the influence of parasitic capacitances and leakage paths that typically degrade the performance of voltage-mode front ends. At the same time, the transformation of the sensor signal into a current allows noise contributions to be shaped differently across the frequency band, potentially improving the overall SNR if properly designed.

Among current-mode building blocks, second generation current conveyors (CCII) and their derivatives have attracted considerable attention in sensor interface applications [110,111,112]. In particular, the second-generation voltage conveyor (VCII) has been proposed as a mixed-mode solution that combines current-domain input processing with a low-impedance voltage output. As the dual counterpart of the well-known CCII, the VCII enables current-mode sensing while directly providing a voltage output compatible with conventional analog and mixed-signal processing stages. Recent studies on VCII-based signal conditioning have highlighted their architectural simplicity, gain programmability, and suitability for low-voltage and low-power operation [113]. Figure 8c shows a VCII-based transimpedance configuration.

Overall, the comparative analysis between current-mode and voltage-mode highlights that conventional voltage-mode and charge-sensitive front ends remain satisfactory reference solutions but operate near their practical limits in low-frequency air-coupled ultrasonic applications. Current-mode and mixed-mode architecture, although more demanding from a design point of view, provides a more flexible and scalable framework for addressing the extreme attenuation, noise dominance, and bandwidth requirements characteristic of ACU systems. These considerations motivate continued research on interface architectures that explicitly leverage current-domain processing and electronics sensor co-design to improve the performance of ACU sensing.

A comparative overview of several state-of-the-art amplifiers and analog interface circuits developed for ACU signal acquisition is presented in Table 7, highlighting the main performance trends emerging from recent literature and emphasizing the differences between voltage-mode and current-mode implementations. While the primary focus of this review is on low-frequency air-coupled ultrasound systems operating approximately in the 20–100 kHz range, Table 7 also includes selected studies at partially higher frequencies to provide a broader technological comparison of interface architectures and system integration strategies. These examples are not intended to redefine the frequency scope of the review, but rather to contextualize the evolution of ACU front-end design.

Importantly, the electronic interface architectures discussed are not intrinsically limited to a specific ultrasonic frequency band. Their operating principles are largely frequency-scalable, with the effective bandwidth being determined by device sizing, biasing conditions, and technology constraints. Therefore, their inclusion contributes to a more comprehensive overview of the state of the art in ACU electronic interfaces, even when demonstrated in slightly different frequency ranges.

### 4.2. Impact of Electronic Interface Architectures on System-Level Performance

Although electronic interfaces are commonly evaluated in terms of gain, bandwidth and noise density, their architectural choices directly determine system-level performance metrics such as minimum detectable displacement, effective sensing range, and achievable signal-to-noise ratio (SNR). For phase-based airborne ultrasonic sensing, $∆x_{min}$ can be approximated as

(1) $$∆x_{min}=\frac{\lambda }{4\pi }\;\frac{v_{n,in}}{A_{sig}}$$

where *λ* is the acoustic wavelength, $v_{n,in}$ is the input-referred RMS noise of the front end, and $A_{sig}$ is the effective demodulated signal amplitude. Therefore, any architectural decision that reduces input-referred noise or stabilizes signal amplitude directly improves displacement resolution. Analytical noise modeling of ultrasonic preamplifiers [107] demonstrates that total input-referred noise depends on transducer impedance, feedback network, and amplifier intrinsic noise. Consequently, impedance matching and feedback optimization directly determine the achievable minimum detectable motion. For capacitive and micromachined ultrasonic transducers, charge-sensitive front ends decouple gain from sensor capacitance and parasitic loading [109], stabilizing signal amplitude under practical packaging constraints. This improves robustness of displacement detectability in real systems. Current-mode approaches based on VCII provide an additional architectural advantage: unlike traditional operational amplifiers, VCII-based transimpedance interfaces allow gain adjustment independently from bandwidth [113]. Experimental results reported for broadband spiral-shaped PVDF sensors demonstrate a flat transimpedance gain of 86 dBΩ over the 20–100 kHz range, with measured sensitivity between −107 dB and −101 dB [116]. This constant-bandwidth behavior ensures that increasing gain does not compress the usable frequency band, thereby preserving both SNR and spectral information important for broadband sonar and biomedical monitoring applications. Receiver integration strategies further influence SNR. Embedded preamplifiers within PVDF receivers amplify weak echoes prior to cable-induced loading and interference, increasing effective signal amplitude relative to downstream noise contributions [114,117]. On the transmission side, high-voltage driver architectures increase acoustic pressure and therefore echo amplitude. Since received pressure decreases approximately with distance and frequency-dependent air attenuation, increased transmitted amplitude directly extends effective sensing range. Finally, bandwidth selection must be co-optimized with system requirements. As summarized in recent preamplifier design reviews [108], increasing gain often requires higher bias current, while integrated noise power scales with receiver bandwidth. Narrowband filtering improves SNR but reduces temporal resolution and time-of-flight precision. In contrast, broadband current-mode architectures such as VCII maintain large bandwidth even at high gain, enabling wideband echo analysis without sacrificing amplitude sensitivity. Table 8 summarizes how specific electronic interface architectures, as reported in the literature, propagate from circuit-level properties to measurable system-level performance metrics in air-coupled ultrasonic systems.

## 5. Signal Processing Strategies

In ACU systems, signal processing is an integral part of the measurement architecture rather than a simple post-processing phase. The high attenuation of ultrasound in air, wavefront dispersion and strong dependence on environmental conditions make the useful signal intrinsically weak and susceptible to distortion, especially in non-invasive biomedical applications. Accordingly, the literature converges on the view that overall performance of ACU systems critically depends on the synergy among sensor design, electronic architecture, and adopted signal processing strategies [118,119,120].

While this system-level perspective is widely acknowledged, the biomedical ACU literature still lacks a structured comparison of post-processing strategies in relation to operating frequency, dominant noise sources, and achievable signal quality. Most contributions address signal processing as an application-specific design choice rather than as a dimension to be analyzed comparatively across studies. Consequently, the impact of different processing approaches on robustness, sensitivity, and system complexity is often implicit and difficult to generalize.

A first distinction emerges when considering the temporal and spatial scale of the physiological phenomena under investigation. For respiratory activity and posture-related movements, which involve slow, large-amplitude movements, TOF estimation and envelope tracking remain the most widely used approaches [44,45,46]. Their prevalence is mainly due to their inherent robustness to amplitude fluctuations, moderate tolerance to environmental variability, and low computational burden. However, the achievable displacement resolution is fundamentally constrained by the ultrasonic wavelength and by the accuracy of TOF estimation under low-SNR conditions, which limits their suitability for fine motion analysis. In contrast, heart-induced chest wall vibrations and subtle biomechanical activities, which target micro-movements, are mainly based on phase demodulation and Doppler-based processing [40,41,42,43,47]. By exploiting phase or instantaneous frequency variations of the received ultrasonic waveform, these techniques enable sub-millimetric sensitivity that cannot be achieved through TOF-based methods. Several studies report reliable heart-rate extraction in fully non-contact configurations, even with light clothing. Nonetheless, this increased sensitivity is accompanied by heightened vulnerability to macroscopic body motion, oscillator phase noise, and thermal drift, thereby imposing stricter constraints on electronic stability, synchronization, and calibration.

To mitigate the severe attenuation and noise inherent to ultrasonic propagation in air, many works adopt coded excitation and correlation-based post-processing schemes. Frequency-modulated chirp signals and coded pulse signals, combined with adaptive filtering methods or pulse compression algorithms, make it possible to increase the transmitted power without violating the constraints of acoustic safety [121,122,123]. These methods have demonstrated a significant improvement in the resistance to detection in a low signal-to-noise ratio, particularly at high frequencies. However, their effectiveness may be compromised by multipath propagation and correlation ambiguities, which necessitate additional signal conditioning or spatial filtering stages.

Spatial processing provides an additional information for post-processing optimization in biomedical ACU systems. Multi-channel averaging and digital beamforming techniques are increasingly employed to enhance directivity, suppress clutter, and stabilize signal quality in the presence of environmental disturbances [41,65]. In array-based configurations, beamforming has been shown to improve the continuity and reliability of respiratory and cardiac estimates, especially in uncontrolled or home-monitoring scenarios. These benefits, however, come with the cost of higher complexity, power, and computation, which could make them unsuitable for wearable or chronic monitoring applications.

A comparative analysis of post-processing approaches reported in the biomedical ACU literature is provided in Table 9. The table highlights the range of operating frequencies, noise types, and types of signal quality improvement provided by post-processing. The key takeaway is that there is not one method that performs best under all circumstances. Instead, a good signal extraction procedure involves a trade-off between micro-motion sensitivity, resistance to environmental/motion noise, and overall system complexity.

The use of machine learning-based approaches introduces non-negligible criticalities, especially in terms of model generalization across different subjects, environmental conditions and hardware configurations. Moreover, the limited interpretability of many data-driven solutions is a critical aspect in the clinical field, where transparency of the decision-making process is often a prerequisite. The trade-off between signal sensitivity, environmental robustness, and implementation complexity is further summarized in Table 10. TOF-Based and Simple Filtering Techniques are on the low-complexity end of the spectrum and are appropriate for embedded designs. Phase- and Doppler-based methods offer the best sensitivity values but require tight control of system stability. Correlation- and multi-channel techniques are more robust but require more hardware resources and computation load.

Finally, post-processing choices are intrinsically linked to the ultrasonic carrier frequency, since attenuation, bandwidth, and achievable displacement resolution in air are strongly frequency dependent. As summarized in Table 11, lower frequencies favor robustness and operating range, whereas higher frequencies enable finer spatial resolution at the cost of increased attenuation and noise susceptibility [31,35].

TOF and envelope tracking mainly capture slow, high-amplitude thoracic motion, supporting respiratory waveform reconstruction and pattern analysis [126]. Phase- and Doppler-based methods provide sub-millimeter sensitivity to cardio-mechanical vibrations, enabling the extraction of heart rate and HRV features relevant to cardiovascular monitoring [127]. Hybrid TOF-phase strategies enable simultaneous respiratory and cardiac tracking with improved robustness, while time–frequency analysis helps separate overlapping physiological components [128,129]. Finally, ML-assisted processing can enhance sensitivity to subtle and non-stationary signatures, thereby potentially supporting early detection of cardiopulmonary or neuromotor alterations. Overall, the choice of processing strategies determines not only signal-domain performance, but also which physiological and clinically relevant parameters can be reliably extracted. Overall, this comparative analysis indicates that signal processing in biomedical ACU systems should be conceived as part of an integrated system-level co-design framework. Post-processing operation can partially compensate for the physical limitations imposed by air propagation, but they cannot fully overcome them. Therefore, optimal performance requires proper integration of frequency selection, sensor and electronic interface design, and processing complexity according to the specific biomedical objective. The first level of processing, dedicated to signal pre-processing, aims to increase the signal-to-noise ratio and stabilize the information acquired before the subsequent analysis stages [130]. A typical pre-processing chain, widely adopted in non-contact biomedical applications, includes bandpass filtering centered on the operating frequency of the transducer, amplitude normalization, and separation of the physiological components of interest. Studies on airborne ultrasonic sensors based on polymeric materials and bio-inspired architectures show how such strategies can significantly reduce environmental interference without compromising the dynamic response of the system [118,131].

In non-contact physiological monitoring applications, such as respiratory activity assessment, low-pass filters are also used to isolate slow signal components, while offset removal and normalization operations are essential to compensate for slow drifts due to electronic instability or environmental variations [132,133]. However, overly selective filtering can result in a loss of temporal information, limiting the system ability to detect rapid changes or physiologically relevant transitions [134]. Time-of-flight (TOF) strategies are one of the most established approaches in ACU systems. An impulse-echo signal, the emitted burst is followed by a delayed echo whose temporal position determines the TOF, while small periodic phase modulations superimposed on the carrier encode micro-displacements, such as chest vibrations induced by the heartbeat. Correlation-based processing further compresses the encoded waveforms, producing a sharp peak that improves temporal localization under low-SNR conditions. TOF estimation enables the calculation of distance variations between the sensor and the target that are directly related to respiratory motion or slow body movements.

TOF methods have been found to be robust to amplitude noise and provide a high level of physical interpretability, thus making them suitable for long-term monitoring in uncontrolled environments [135]. Additionally, differential TOF analysis helps to compensate for changes in the speed of sound in ambient air due to variations in temperature and humidity, which is very important in biomedical applications [132,133]. However, there remains an intrinsic limitation related to reduced sensitivity to micro-movements, which limits its use to respiratory monitoring rather than cardiac activity detection. To overcome these limitations, numerous studies propose the analysis of the phase of the ultrasonic signal or the use of Doppler techniques, capable of detecting sub-millimeter micro-movements with high sensitivity. Compared to TOF techniques, phase analysis offers significantly higher spatial resolution, making it possible to detect cardiac activity even in completely non-contact configurations [119,120]. However, these systems involve increased complexity in electronic systems and are highly sensitive to macroscopic motions of the body.

The need for coherent demodulation and the presence of oscillators impose a major constraint in terms of system calibration. At the same time, strategies based on the use of modulated signals and correlation techniques have been explored to improve the signal-to-noise ratio in conditions of strong attenuation. The use of coded pulses or chirp signals allows the transmitted energy to be increased while keeping the peak acoustic pressure limited, a particularly important requirement in the biomedical field. Studies on airborne ultrasonic systems show that these approaches are more robust than simple pulses, especially in complex indoor environments [135]. However, the presence of multipath phenomena can generate ambiguity in the correlation function, requiring additional processing stages and increasing the overall computational load. A further evolution of signal processing strategies in ACU systems is represented by multi-channel processing and digital beamforming.

The conceptual diagram of an airborne ultrasonic array with digital beamforming for non-invasive biomedical applications demonstrates how spatial processing improves the directivity of the system, reducing the impact of unwanted reflections and increasing the stability of physiological estimates, while supporting multi-target applications [118,120]. However, these advantages are accompanied by a significant increase in hardware complexity and power consumption, limiting their use in wearable devices or long-term home monitoring systems. In recent years, ACU signal processing strategies have been further improved through integration with data-driven approaches and artificial intelligence systems. In this context, traditional signal processing is often used to extract physically interpretable features, which are then processed using machine learning models to improve the accuracy and robustness of the system [136,137]. Despite the high potential, the literature highlights critical issues related to model generalization, dependence on training data, and limited interpretability of decisions, aspects of relevance in the clinical setting.

A comparative summary of the main signal processing strategies adopted in ACU systems is shown in Table 12, which shows that there is no universally optimal solution, but rather a compromise between sensitivity to micro-movements, environmental robustness, and computational complexity, which must be evaluated according to the specific application. The comparative analysis shown in Table 12 clearly highlights how the selection of signal processing strategies in ACU systems should be guided primarily by the trade-off between sensitivity to micro-movements, environmental robustness and computational complexity, rather than by the search for a universally optimal solution.

Filtering and pre-processing techniques, while representing the minimum necessary level in any ACU architecture, are inherently limited in terms of sensitivity and cannot, on their own, support applications that require the detection of complex physiological micro-movements. In contrast, TOF-based approaches show a favorable combination of environmental robustness and simplicity of implementation, making them particularly suitable for long-term respiratory monitoring in uncontrolled environments, but less effective for cardiac applications. Strategies based on phase analysis and Doppler techniques emerge as the most suitable for detecting sub-millimeter micro-movements, as confirmed by the work reported in [119,120]. On one side, high sensitivity is balanced by a larger vulnerability with respect to macroscopic motions and a requirement for system stability and calibration. On the other side, the use of modulated signals and correlation processing provides a larger robustness with respect to noise and attenuations but also provides ambiguities with respect to multipaths and a complexity increase that need to be balanced with hardware capabilities.

Multi-channel beamforming solutions are the most comprehensive solution from a performance perspective. They provide very good sensitivity with robustness to environmental changes. Nevertheless, as seen from Table 9, this solution incurs maximal complexity with respect to hardware and energy. Thus, this solution is best suited for fixed installations rather than wearable systems and home monitoring. Finally, hybrid approaches that integrate traditional signal processing and artificial intelligence models are a promising direction for overcoming the limitations of individual techniques. The possibility of combining physically interpretable features with data-driven models allows for improved system sensitivity and robustness, as shown in [136,137]. However, the adoption of such approaches in the clinical setting requires careful evaluation of model generalizability, decision transparency, and computational sustainability, aspects that still represent an open challenge in the design of reliable and clinically validated ACU systems. In this context, recent studies increasingly highlight the role of artificial intelligence not only as a final classification or regression tool, but as an advanced post-processing stage for airborne ultrasonic signals. In such approaches, pre-processed ACU signals are fed into machine learning algorithms. These signals can be converted into time-frequency domain signals so that the machine learning algorithms can address issues like nonlinear attenuation, multipath, and environment introduced when ultrasound signals travel through air. These data-driven post-processing techniques enable the extraction of robust physiological patterns even under low signal-to-noise ratio conditions. Although several methodological advances have initially been developed in the broader field of non-contact sensing, including microwave-based vital sign monitoring, the underlying signal processing and learning paradigms are directly transferable to ACU systems. Recent review studies demonstrate that machine learning-based post-processing significantly improves the stability and accuracy of vital-sign estimation in dynamic and uncontrolled conditions, compared to purely deterministic signal processing pipelines [136]. This is particularly important in the case of ACU systems implemented in practical biomedical applications, where variability is difficult to control.

A further trend that is currently emerging is the incorporation of artificial intelligence directly after ultrasonic signal processing pipelines. Advances in intelligent nano- and micro-scale sensors and actuators indicate that the combination of physically interpretable ultrasonic features with embedded learning models can support local decision-making and edge-level processing, thereby reducing latency and dependence on external computational resources [137]. In this framework, conventional signal processing retains a fundamental role in ensuring physical interpretability, while artificial intelligence provides higher-level robustness and adaptability.

Nevertheless, the literature consistently notes that AI-based post-processing of air-coupled ultrasonic signals still faces significant challenges. Key issues include the availability of representative training datasets, the generalization capability of learned models across different subjects and environmental conditions, and the limited interpretability of model outputs. These problems become even more relevant in a clinical setting where transparency, accuracy, and validation are crucial. Future research will focus on the development of hybrid solutions balancing accuracy and understandability.

## 6. Discussion

This review highlighted how low-frequency ACU is being consolidated as a fully non-contact sensing modality in two converging biomedical domains: (i) contactless monitoring of physiological micro-movements (respiration and cardio-mechanical activity) and (ii) medical/assistive robotics and human–machine interfaces requiring short-range ranging, tracking, and coarse geometric reconstruction. The reviewed ACU systems can be compared using five key metrics: operating frequency, detection range, minimum detectable displacement/pressure, achievable signal SNR, and overall complexity. A clear trade-off emerges: lower frequencies (<60 kHz) reduce atmospheric attenuation and therefore extend range, whereas higher frequencies (>60 kHz) improve displacement resolution but suffer higher absorption in air. Multi-channel arrays can recover sensitivity and improve robustness through spatial averaging and beamforming, but at the cost of increased hardware and computational complexity. Overall, the framework highlights a fundamental performance frontier in which long-range operation favors <60 kHz, while sub-millimetric resolution for cardiac monitoring typically requires higher frequencies, with complexity becoming a strategic lever rather than a mere constraint. Across these domains, the primary performance bottleneck is not the sensing principle itself, but the system-level co-design needed to reliably extract weak echoes in air under realistic environmental and motion variability [17,18,21]. From a physics perspective, the pronounced acoustic-impedance mismatch at the transducer–air interface, together with the frequency-dependent air attenuation, imposes an intrinsic trade-off among operating range, spatial resolution, and robustness [32,33]. In the 20–100 kHz band, practical systems typically favor lower-frequency operation to extend range and mitigate attenuation, while relying on waveform engineering (bursts, chirps, coded sequences) and coherent processing (phase/Doppler) to regain sensitivity to sub-millimetric motion [40,41,42,43,44,121,122]. Temperature and humidity dependence, multipath propagation, and clutter further motivate calibration and compensation strategies and, where feasible, spatial filtering via multichannel or array-based acquisition [41,65]. Sensor technologies are consequently evolving along two complementary directions. Horn-loaded or matched PZT transducers remain attractive for high-SPL transmission and longer-range operation, but their narrowband response and bulk can limit wearable/embedded integration. Piezopolymer solutions (PVDF and related ferroelectric/ferroelectret architectures) offer broadband operation and more favorable acoustic impedance and have proven particularly effective as receivers when paired with ultra-high input impedance, low-noise interfaces [18,24,107]. MEMS-based CMUT/PMUT arrays, in turn, enable dense multi-channel architectures and electronic beamforming to stabilize performance in cluttered scenes, albeit often at the cost of increased system complexity and stringent drive and interface requirements [22,103,104]. Concerning electronic interfaces, receive chain—especially the first preamplifier stage—constitutes the main system bottleneck (not a secondary “conditioning” block): small improvements in input noise or impedance matching can yield system-level gains comparable to transducer optimization. Since voltage/charge approaches operate near practical limits in low-frequency ACU, current-mode and mixed-mode interfaces (e.g., CCII/VCII-based transimpedance) are increasingly attractive: they can avoid GBW constraints, better tolerate parasitic components via low-impedance input nodes, and reshape noise for improved SNR—at the cost of more demanding design. Along the paper, we have highlighted competitive sensitivity and simple, low-power VCII/TIA solutions in the 20–100 kHz range alongside established voltage/charge implementations at higher frequencies. On the algorithmic side, TOF/envelope tracking generally provides robust respiration monitoring, whereas phase/Doppler processing is required for cardiac micro-motion but demands higher stability and motion-artifact handling. Hybrid pipelines and adaptive filtering can help bridge these operating regimes [40,41,42,43,44,45,46,47,48,49]. To provide a quantitative metric for comparison, the literature reported in Table 1 evidenced that, in vibrocardiography, a direct comparison between standard ECG and LVCG shows a mean error of approximately 0.09 ± 5.54 ms when estimating V–V and R–R intervals [40]. Regarding respiratory-rate monitoring, the respiration count measured using a nasal thermocouple sensor was compared with that obtained using the US system. Across all subjects, the correlation coefficient was 0.982 for unclothed measurements and 0.939 for clothed measurements. The difference between the two sensing methods resulted in a mean difference of 0.006 ± 0.294 breaths/min on bare skin and 0.002 ± 0.269 breaths/min when clothing was worn. For respiratory-waveform estimation, the comparison was carried out using the normalized mean squared error (NMSE), which remained below 1% [41]. For HRV evaluation, the error, defined as the difference in R–R peak locations between standard ECG and the US-based system, was 30.87 ms, while the root mean square of successive differences (RMSSD) was 22.74 ms [43]. This value is slightly above the 20 ms threshold, often considered clinically acceptable. HR monitoring based on multichannel ultrasound was validated against an ECG reference using Bland–Altman analysis [42]. The mean absolute error (MAE) ranged from 14 × 10^−3^ to 35 × 10^−3^ bpm, with a standard error (SE) between 1.6 × 10^−3^ and 6.9 × 10^−3^ bpm [42]. Concerning the application of ACUs for gesture identification, as those reviewed in Table 2, an average accuracy of 74.24% and a median accuracy of 75.76% were obtained, showing better performance than some approaches but worse than others [55]. For instance, an ultrasound Doppler system combined with a Gaussian Mixture Model (GMM) achieved 88% accuracy. Another system coupled a single piezoelectric transducer with an eight-element microphone array and deep-learning methods, with accuracy values ranging from 64.50% to 96.90%. Moreover, a four-channel A-mode ultrasound device achieved an overall accuracy of 77.43%, which improved to 80.21% when ultrasound was combined with surface electromyography (sEMG). In a study on a human ultrasonic echolocation device, performance comparison was made between a commercial sensor and a sensor modified with Differential Phase Shift Keying (DPSK) modulation [7]. Without modulation, 55 errors occurred over 692 measurements, corresponding to an error rate of 7.95%, while with modulation, the error rate dropped to 1.01%. As for smart glasses, localization accuracy was assessed using confusion-matrix analysis in narrow (45°) and wide-angle (90°) scenarios: in the wide-angle case, accuracy ranged from 80 to 100% at the extremes and was about 85% at the center, whereas in the narrow-angle case it was ~80% at the extremes and 40–60% at the center [57]. Finally, the smart cane system was validated by comparing the distance measured by the ultrasonic sensors with the actual distance measured using a ruler [58]. The proposed model achieved an average accuracy of 98%, with an average error rate below 2%, demonstrating reliable obstacle-detection performance. The comparative evidence indicates that there is no universally “best” ACU architecture: the optimal design is strongly task-dependent. For high-sensitivity, contactless vital-sign monitoring (roughly in the 20–100 kHz range), the most effective overall scheme is the integration of curved PVDF receivers with current-mode VCII-based transimpedance amplifiers, because this configuration maximizes SNR while mitigating the bandwidth (GBW) and parasitic limitations typical of voltage-mode front ends. Conversely, for applications such as medical robotics, short-range spatial tracking, and human–machine interaction—where spatial selectivity, miniaturization, and geometry reconstruction are key—MEMS CMUT/PMUT array architectures with electronic beamforming emerge as the superior option, despite higher complexity and power demand. Across the surveyed studies, several ACU solutions achieve performance comparable to clinical or instrumental references: near-zero biases with millisecond-scale dispersion for interval estimation, high correlations in respiratory monitoring, NMSE below 1% for waveform estimation, strong agreement for HRV metrics relative to ECG, and excellent ECG–ultrasound concordance in multichannel cardiac monitoring. In assistive/HMI use cases, reported accuracy typically spans from ~74% to ~98%, with notable gains enabled by DPSK modulation, Doppler approaches, and multimodal sensing; moreover, localization accuracy can vary substantially with the tested scenario (e.g., field-of-view angle in smart-glasses systems).

## 7. Conclusions

Despite rapid progress, the surveyed literature remains heterogeneous in reporting practices, which hinders reproducibility and cross-study comparison. To support biomedical translation, a minimum reporting set should consistently include: the operating frequency band and excitation waveform; emitted SPL and reference distance; receiver sensitivity and calibration procedure; directivity/beamwidth; complete front-end schematic or equivalent parameters (input impedance, gain, bandwidth, input-referred noise); digitization settings; environmental conditions (temperature/humidity) and compensation; and evaluation protocols/metrics (e.g., RR/HR error under controlled motion and through-clothing conditions). Establishing shared benchmarking datasets and test scenarios would further accelerate objective comparison of sensors, interfaces, and processing strategies. The evolution of ACU systems for biomedical applications is expected to progress through distinct technological stages. Looking forward, the most impactful advances in the short term (1–3 years) are expected to come from co-design approaches that jointly optimize transducer geometry/materials, low-noise interfaces, and application-aware processing. Emerging directions include flexible and large-area airborne arrays, multimodal fusion with optical/radar/inertial sensing to improve robustness, and interpretable AI-assisted post-processing for operation in low-SNR, multipath-rich environments. Overall, low-frequency ACU is well positioned to enable privacy-preserving, contactless biomedical monitoring and assistive technologies; achieving clinical-grade reliability will require standardized validation workflows and system-level design rules that are portable across devices and deployment settings. In the medium-term evolution (3–5 years), future research on low-frequency airborne ultrasonic contactless systems is expected to evolve along a few main directions. Further improvements will likely stem from tighter co-design of the acoustic front end and the electronic interface. In particular, the development of PVDF-based curved or structured receivers specifically tailored to current-mode transimpedance architectures may further enhance SNR without increasing acoustic power. As voltage- and charge-based interfaces approach practical limits in low-frequency ACU, current-mode solutions (e.g., VCII/CCII-based architectures) represent a promising pathway. Future efforts should therefore focus on low-power CMOS implementations, noise-shaping strategies, and improved robustness to parasitic capacitances to enable wearable and embedded deployments. In parallel, the integration of adaptive filtering, model-based estimation, and lightweight machine learning techniques could significantly improve resilience to motion artifacts, multipath effects, and environmental variability. Hybrid time-of-flight/phase and Doppler processing pipelines may support seamless operation across respiratory and cardiac monitoring regimes. For robotics and human–machine interfaces, future developments will likely emphasize compact MEMS array platforms with integrated beamforming and sensor fusion (e.g., ultrasound combined with inertial or optical sensing) to improve reliability in dynamic environments. In the longer term (>5 years), the convergence of optimized piezopolymer receivers, ultra-low-noise current-mode electronics, and adaptive processing may enable fully wearable, calibration-light, and clinically robust non-contact systems suitable for continuous healthcare applications. At this stage, ACU systems would transition from experimental sensing platforms to validated, non-contact diagnostic infrastructures. This evolution is embedded within healthcare environments, through distributed ACU sensing integrated in smart environments (e.g., hospital rooms, assisted-living facilities, rehabilitation centers), autonomous AI-driven interpretation frameworks capable of detecting early pathological signatures from multimodal ultrasonic micro-movements patterns, possible development of standardized ACU clinical biomarkers for cardiopulmonary and neuromotor assessment and their integration into assistive robotic systems capable of combining physiological monitoring with spatial perception.

## Figures and Tables

**Table 1:** Contactless ACU studies for physiological monitoring.

Application	Operating Mode	Transducers (Material/Type)	Frequency Range (kHz)	Bandwidth	Sensitivity	Electronic Interfaces	SPL* (dB)	Comparison	Ref.
Vibrocardiography (HR)	Pulse-Wave Doppler	PZT 20-1330 (APC International Ltd., Burlingame, USA) transducer + ultrasonic microphone FG-23329, Knowles Electronics receiver	20–60 (operating frequency), 50 (carrier frequency PW Doppler)	20–60 kHz (Emission-reception system), 10–100 kHz (electronic preamplifier)	−53 dB	Agilent U2542A DAQ + microphone, preamplifier	N/A	0.09 ± 5.54 ms V-V vs. R-R interval	[40]
Respiratory Rate Monitoring	TOF	Ultrasonic proximity sensor (integrated Tx/Rx)	240 (proximity sensor)	N/A	N/A	0–10 V DC analog output + BIOPAC MP150 DAQ	N/A	0.006 ± 0.294 breaths/min (bare) 0.002 ± 0.269 breaths/min (clothing)	[46]
Respiratory Waveform Estimation	Pulsed excitation: pulsed Doppler; FMCW: FMCW Doppler	Emitter: 5 W speaker; Receiver: 4 × 4 MEMS array (UMA-16, SPH1668LM4H)	Pulsed: 18; FMCW: 16.8–20.8; Beamforming pulses: 8	4 kHz (16.8–20.8 kHz)	−29 dB	Microphone array + amplification + ADC (44.1 kHz) + digital processing	120	US vs. IMU, error < 1%	[41]
Air-ultrasound skin motion for heart rate and heart rate variability (HR/HRV)	Air-ultrasound transducer + motion tracking (TOF air-ultrasound distance measurement)	Air-coupled piezoelectric ultrasound transducers (Multicomp Pro MCUSD40A100B17RS)	~100 (operating frequency), 95–105 (chirped excitation)	89–111 kHz (transducer bandwidth, 22 kHz −6 dB electroacoustic response)	N/A	GRAS^TM^ 12AA 2-channel power module (high-voltage waveform generator, impedance matching circuit, transceiver, digitizer)	110–125	ECG vs. US, 30.87 ms (in R–R peak) with an error on successive differences of 22.74 ms	[43]
Chest vibrometry (RR)	TOF/phase-delay; cross-correlation of successive echoes, measuring surface normal velocity	Emitter: 37 piezoelectric diaphragms (Murata 7BB-20-6), all in parallel for ultrasonic emission, Receiver: 6 high-frequency microphones (Knowles FG-23329) connected in parallel	20–60	40 kHz	−53 dB each	Piezoelectric array driven by single electronic amplifier (emission) + microphone signal 40 dB amplified, analog front-end+ Agilent U2542A DAQ	100–115	N/A	[6]
Multi-channel ultrasound system for HR monitoring	CW ultrasound Doppler (phase-based)	Piezoelectric US (SensComp 40LT16 transmitter/40 LR 16 receiver)	40	2 kHz@−6 dB	−65 dB	VCO + phase detector (XOR) + LPF + ADC	120	ECG vs. US, mean absolute error of 14 × 10^−3^−35 × 10^−3^ bpm, and a standard error of 1.6 × 10^−3^−6.9 × 10^−3^ bpm	[42]

* SPL: Sound pressure level.

**Table 2:** Contactless ACU studies for physiological and motor applications.

Application	Operating Mode	Transducers (Material/Type)	Freq. (kHz)	Band (kHz)	Sensitivity	SPL	Electronic Interfaces	Comparison	Refs.
Hand gesture/person identification	Pulse-echo/time-of-flight	HC-SR04 ultrasonic distance sensor module	40	1–3	−65/−75 dB	110–120 dB	Arduino Mega 2560 + ATtiny85;	Accuracy of 74.24%	[55]
Human ultrasonic echolocation device	Pulsed Frequency-Modulated (FM) ultrasonic chirps	Ultrasonic loudspeaker (Tx) Fostex FT17H Realistic Super Tweeter + 2 Ultrasonic microphones (Rx, binaural) Bruel & Kjaer Type 4939 microphones	5–50	45	4 mV/Pa	98.5 dB/W	PC-based signal generation and processing (MATLAB) + Sound card ESI Juli@ (192 kHz I/O) + Power amplifier Lepai Tripath TA2020 + Microphone preamplifier B & K 2670 + B & K Nexus + Playback: Gigaport HD USB sound card + open-ear headphones	N/A	[62]
Obstacle detection for visually impaired (assistive)	Pulsed ultrasonic TOF with phase-modulated pulse trains (DPSK); distance calculation via TOF + echo validation via phase-code modulation	2 × piezoelectric air-coupled ultrasonic transducers per sensor (1 Tx + 1 Rx), SRF08, HC-SR04	40	1–3	−65/−75 dB	110–120 dB	Ultrasonic module: HC-SR40 (modified) + microcontroller ATMega328P (Arduino Nano) + digital trigger/echo interface + on-board analog comparator and ADC for echo detection + Bluetooth module (HC-06) for data transmission to smartphone	Error of 7.95% without phase modulation. Error of 1.01%, with phase modulation	[7]
Smart glasses for blind people	Frequency modulation/demodulation	Omni-directional digital microphones (IMP34DT05TR)	20–48	N/A	N/A	N/A; <70 dB (environment)	Digital audio interface (PDM, I^2^S)	2 samples delay	[57,60]
Smart cane	TOF	HC-SR04 ultrasonic transceiver	40	N/A	N/A	N/A	Digital I/O (TRIG/ECHO timing interface)	Accuracy of 98%, and avg. error < 2%	[58,59]

**Table 3:** Material for ACU with their functional roles, advantages and limitations.

Material	Acoustic Characteristics	Advantages	Limitations	Refs.
PZT	High piezoelectric coefficients; acoustic impedance of 30–35 MRayl	Strong electromechanical coupling; efficient emission and reception; mature fabrication technology	Severe impedance mismatch with air; requires matching layers; narrow bandwidth	[34,35]
Composites (PZT/polymer)	Acoustic impedance of 10–15 MRayl; enhanced compliance	Improved impedance matching in air, broader bandwidth; higher sensitivity in air	Lower mechanical robustness; more complex manufacturing	[27]
PVDF	Low acoustic impedance (3–4 MRayl); high piezoelectric flexibility	Lightweight, flexible, and suitable for airborne coupling; good SNR when designed properly	Lower electromechanical coupling vs. ceramics; higher electrical noise	[36]
Polypropylene Foams	Ultra-low acoustic impedance (0.05–0.1 MRayl); internal charged voids act as piezoelectric domains	Excellent acoustic matching to air; can act simultaneously as active transduction and matching layer; low mass	Limited power handling; potential aging of charged voids	[23,37]
Silicone Rubber Porous/Semiporous Membranes	Tunable acoustic impedance via air-filled microstructures; support half-wavelength cavity resonance	Enables highly efficient acoustic emission into air using resonance; adaptable to many transducer geometries	Narrowband response; sensitive to manufacturing tolerances	[23]
Aerogel	Extremely low density, acoustic impedance near air (0.02–0.03 MRayl)	Near-ideal impedance match; high transmission efficiency; emerging interest for broadband ACU	Fragile microstructure; challenging fabrication: moisture sensitivity	[35]
Silicone rubber	Reduced effective impedance due to micro voids	Improves transmissivity between transducer and air; simple and inexpensive	Limited bandwidth; material aging; strongly frequency-dependent	[81,82]

**Table 4:** Summary of PVDF in-air ultrasonic sensor geometries.

Geometry	Frequency (kHz)	Bandwidth (kHz)	Directivity	Sensitivity (dB)	Applications	Ref.
Cylindrical	40, 80	8, 10	H: 360 V: ±40	−76, −90	Positioning, ranging	[18]
Hemi-cylindrical	30–65	35	H: − V: ±15	−52	Obstacle detection	[18]
Semi-conical	24–36	12	H: ±50 V: ±60	N/A	Robotic sensing	[75]
Truncated conical	25–36	11	H: 360 V: ±70	N/A	3D positioning	[75]
Spiral-shaped	30–95	~60	H: 360 V: 360	H: −89.1 to −96.1 V: −94.2 to −103.8	Biomimetic sonar	[96]
Quasi-spherical	30–50	20	H: 360 V: ±120	N/A	Localization	[18]

**Table 5:** Summary of MEMS ultrasonic sensor geometries.

Technology	Geometry	Materials	Dimensions	Frequency Range (kHz)	Fractional Bandwidth *	SPL	Ref.
CMUT	membrane	Si/SiN	≈32 × 32 µm^2^; ≈250 nm gap	20–100	wide (up to ≈100–175% fractional BW)	≈82 dB @ 40 kHz (8.9 cm)	[104]
PMUT	membrane	PZT or AlN/ScAlN	tens–hundreds µm	≈40	moderate (≈55% fractional BW, limited by residual stress)	100.3 dB @ 40 kHz (33 cm); >120 dB @ 10 cm (array)	[103]

* Fractional bandwidth (FBW) = Δf/f_c_, where Δf is the difference between the upper and lower −6 dB frequencies, and f_c_ is the center frequency. It represents the −6 dB bandwidth of the transducer response, normalized to its center frequency (typically expressed in percentage).

**Table 6:** Comparative overview of air–solid acoustic matching strategies and technical advantages across the main ACU sensor technologies.

Sensor Technology	Mismatch Solutions	Advantages	Drawbacks
Piezoceramics	λ/4 horn as mechanical impedance transformer;	High SPL; long-range transmission	Narrowband; bulky; strong intrinsic Z mismatch
Piezopolymers	Lower intrinsic Z; compliant/curved geometries; optional ultra-low-Z front layers and tuned backing	Broadband; lightweight and conformal; effective as receiver	Lower coupling and SPL; high source impedance; needs high-Z, low-noise electronics
MEMS	Micromachined membrane + cavity tuning; packaging as acoustic window; array gain/beamforming	Miniaturization; dense arrays; electronic beam steering	Limited power; bias (CMUT); packaging and crosstalk complexity

**Table 7:** Summary of state-of-the-art electronic interfaces for low-frequency ultrasonic sensors.

Transducer	Front End	Frequency Range	Noise or Sensitivity Focus	Key Advantages	Ref.
Air-coupled piezoelectric	Voltage-mode low-noise preamplifier	400–800 kHz	Analytical SNR optimization	Rigorous noise modeling including transducer	[107]
AlN PMUT array	Voltage vs. charge amplifier (CMOS)	~3 MHz (liquid)	VA shows superior SNR; input noise of 0.08 pA/√Hz (VA) vs. 0.15 pA/√Hz (CSA)	CMOS integration, low power, removal of crowbar current, and reduced parasitic elements	[109]
PVDF hydrophone	Integrated voltage preamplifier	100 kHz–1.5 MHz	High sensitivity PVDF receiver (1.62 V/MPa)	MRI compatibility and low cost	[114]
Logarithmic spiral-shaped PVDF	VCII-based TIA	20–80 kHz	Sensitivity ≈−100 dB	Low power consumption (6 mA), simple single-stage architecture, bypasses GBW constraints	[115]
PVDF spiral ACU	VCII-based TIA + filter	20–100 kHz	Sensitivity comparable to commercial sensors (−120 to −92 dB)	Bio-inspired design (mammalian cochlea), 360° omnidirectional pattern, easy fabrication	[116]
Miniature PVDF (110 µm)	Unity gain preamplifier (LMH6639 Op-Amp)	0.51 MHz–5.4 MHz	High sensitivity (2.36–3.87 V/MPa); noise floor of 0.21 kPa at 1.1 MHz	Extremely low cost (<4 USD), wide acceptance angle (54° at 1.1 MHz), and subharmonic detection	[117]

**Table 8:** Relationship between electronic interfaces and system-level performance metrics.

Front-End	Key Circuit Property	Direct System-Level Impact	Ref.
Voltage-mode low-noise preamplifier	Reduced input-referred noise via impedance and feedback optimization	↓ Minimum detectable displacement, ↑ SNR	[107]
Voltage vs. charge amplifier (CMOS)	Gain independent of sensor capacitance, the VA–CSA choice affects SNR through both the input-referred noise and the effective signal transfer	Stable signal amplitude, ↓ sensitivity to parasitic components	[109]
Integrated voltage preamplifier	Local preamplification close to sensing element	↑ Effective SNR, reduced cable-induced degradation	[114]
VCII-based TIA	Gain independent from bandwidth (current-mode processing)	Wide bandwidth with reduced architectural complexity	[115]
Broadband PVDF + VCII sonar system	Flat 86 dBΩ transimpedance	Broadband sensing, improved SNR with reduced parasitic sensitivity	[116]
Unity gain preamplifier (LMH6639 Op-Amp)	Wideband sensitivity with spatial mapping capability, the front-end noise sets the lowest detectable emission level	↑ Spectral detectability, improved cavitation monitoring accuracy	[117]

**Table 9:** Post-processing strategies for ACU in biomedical applications.

Application Domain	Post-Processing Technique	Typical Frequency Range	Dominant Noise Source	Signal Quality Improvement	Refs.
Respiration monitoring	TOF/envelope tracking	20–40 kHz	Environmental noise, drift	Robust distance estimation	[41,45,46]
Cardiac monitoring	Phase/Doppler analysis	~40 kHz	Motion artifacts, phase noise	Sub-mm displacement sensitivity	[40,41,42,46]
Vital signs (HR + RR)	Hybrid (TOF + phase/Doppler)	~40 kHz	Mixed: macroscopic body motion, drift, phase noise, multipath	Simultaneous HR/RR extraction; improved stability vs. single domain (TOF for large motion, phase for micro-motion)	[48,49]
Low-SNR sensing	Chirp + matched filtering	30–90 kHz	Mixed (motion + electronic)	Improved stability vs. single domain	[121,122]
Multi-target sensing	Beamforming/array processing	40–80 kHz	Attenuation, broadband noise	Spatial filtering and robustness	[41,65]
Feature extraction	ML-assisted post-processing	Application-dependent	Non-stationary noise	Improved estimation robustness	[123,124,125]

**Table 10:** Trade-off between sensitivity, robustness, and complexity in ACU signal processing.

Processing Strategy	Sensitivity to Micro-Movements	Environmental Robustness	Hardware Complexity	Computational Complexity
Basic filtering	Low–Medium	Medium	Low	Low
TOF tracking	Medium	High	Low	Low
Chirp/correlation	High	Low	Medium	Medium–High
Beamforming/arrays	High	High	High	Very high
Hybrid DSP + AI	Very high	Variable	High	Very high

**Table 11:** Relationship between ultrasonic frequency range and signal quality in air-coupled biomedical ultrasound.

Frequency Range	Typical Applications	Preferred Post-Processing	Main Limitations
20–30 kHz	Respiration, gross motion	TOF, envelope	Limited spatial resolution
~40 kHz	Vital signs, cardiac monitoring	Phase/Doppler, hybrid	Sensitivity to motion artifacts
60–100 kHz	Fine motion, arrays	Chirp, beamforming	Strong air attenuation

**Table 12:** Comparison between the main signal processing strategies in ACU systems.

Strategy	Sensitivity to Micro-Movements	Environmental Robustness	Complexity	Refs.
Filtering and pre-processing	Low–Medium	Medium	Low	[132,133]
TOF	Medium	High	Low	[132,133,135]
Phase/Doppler analysis	High	Low	Medium–High	[119,120]
Correlation and modulated signals	Medium–High	Medium	High	[135]
Multi-channel beamforming	High	High	Very high	[120]
Hybrid approaches with AI	Very high	Variable	Very high	[136,137]

## Data Availability

No new data were created or analyzed in this study. Data sharing is not applicable to this article.

## References

[1] Hassanpour A., Yang B. (2025). Contactless Vital Sign Monitoring: A Review Towards Multi-Modal Multi-Task Approaches. Sensors.

[2] Al-Naji A., Al-Askery A.J., Gharghan S.K., Chahl J. (2019). A System for Monitoring Breathing Activity Using an Ultrasonic Radar Detection with Low Power Consumption. J. Sens. Actuator Netw..

[3] Kabiri M., Cimarelli C., Bavle H., Sanchez-Lopez J.L., Voos H. (2022). A Review of Radio Frequency Based Localisation for Aerial and Ground Robots with 5G Future Perspectives. Sensors.

[4] Tsao J.Y., Crawford M.H., Coltrin M.E., Fischer A.J., Koleske D.D., Subramania G.S., Wang G.T., Wierer J.J., Karlicek R.F. (2014). Toward smart and ultra-efficient solid-state lighting. Adv. Opt. Mater..

[5] Madore B., Preiswerk F., Bredfeldt J.S., Zong S., Cheng C.-C. (2021). Ultrasound-based sensors to monitor physiological motion. Med. Phys..

[6] Niérat M.-C., Laveneziana P., Dubé B.-P., Shirkovskiy P., Ing R.-K., Similowski T. (2019). Physiological Validation of an Airborne Ultrasound Based Surface Motion Camera for a Contactless Characterization of Breathing Pattern in Humans. Front. Physiol..

[7] Abreu D., Toledo J., Codina B., Suárez A. (2021). Low-Cost Ultrasonic Range Improvements for an Assistive Device. Sensors.

[8] Qiu Z., Lu Y., Qiu Z. (2022). Review of Ultrasonic Ranging Methods and Their Current Challenges. Micromachines.

[9] Diebold C.A., Salles A., Moss C.F. (2020). Adaptive Echolocation and Flight Behaviors in Bats Can Inspire Technology Innovations for Sonar Tracking and Interception. Sensors.

[10] Marzo A., Corkett T., Drinkwater B.W. (2018). Ultraino: An Open Phased-Array System for Narrowband Airborne Ultrasound Transmission. IEEE Trans. Ultrason. Ferroelectr. Freq. Control.

[11] Wu Q., Chen Q., Lian G., Wang X., Song X., Zhang X. (2021). Investigation of an air-coupled transducer with a closed-cell material matching strategy and an optimization design considering the electrical input impedance. Ultrasonics.

[12] Fiorillo A.S. (2000). Noise analysis in air-coupled PVDF ultrasonic sensors. IEEE Trans. Ultrason. Ferroelectr. Freq. Control.

[13] Kelly S.P., Hayward G., Gomez T.E. (2001). An air-coupled ultrasonic matching layer employing half wavelength cavity resonance. 2001 IEEE Ultrasonics Symposium. Proceedings. An International Symposium (Cat. No.01CH37263), Atlanta, GA, USA.

[14] Xu J., Ye Y., Dong T., Yang Z., Pires N.M.M., Zhou Y., Tao F., Wang J., Zhang J., Luo G. (2025). State of the Art of Low-Frequency Acoustic Modulation: Intensity Enhancement and Directional Control. Adv. Sci..

[15] Wang S., Mei L., Liu R., Jiang W., Yin Z., Deng X., He T. (2024). Multi-modal fusion sensing: A comprehensive review of millimeter-wave radar and its integration with other modalities. IEEE Commun. Surv. Tutor..

[16] Redij R., Kaur A., Muddaloor P., Sethi A.K., Aedma K., Rajagopal A., Gopalakrishnan K., Yadav A., Damani D.N., Chedid V.G. (2023). Practicing Digital Gastroenterology through Phonoenterography Leveraging Artificial Intelligence: Future Perspectives Using Microwave Systems. Sensors.

[17] Chimenti D.E. (2014). Review of air-coupled ultrasonic materials characterization. Ultrasonics.

[18] Pullano S.A., Critello C.D., Bianco M.G., Menniti M., Fiorillo A.S. (2021). PVDF ultrasonic sensors for in-air applications: A review. IEEE Trans. Ultrason. Ferroelectr. Freq. Control.

[19] Zhang Y., Jin T., Deng Y. (2024). A low-voltage-driven MEMS ultrasonic phased-array transducer for fast 3D volumetric imaging. Microsyst. Nanoeng..

[20] Peng X., Hu L., Liu W., Fu X. (2020). Model-Based Analysis and Regulating Approach of Air-Coupled Transducers with Spurious Resonance. Sensors.

[21] Rathod V.T. (2020). A Review of Acoustic Impedance Matching Techniques for Piezoelectric Sensors and Transducers. Sensors.

[22] Lin J.L., Kao C.L., Wu S.W., Hsu H.J., Lin H.C., Li C.Y., Chen C.Y., Huang C.H. (2025). Air-coupled piezoelectric micromachined ultrasonic transducer array based on low-cost and large remnant polarization PZT thin film. Sci. Rep..

[23] Demi L. (2018). Practical Guide to Ultrasound Beam Forming: Beam Pattern and Image Reconstruction Analysis. Appl. Sci..

[24] Xin Y., Sun H., Tian H., Guo C., Li X., Wang S., Wang C. (2016). The use of polyvinylidene fluoride (PVDF) films as sensors for vibration measurement: A brief review. Ferroelectrics.

[25] Van Neer P.L.M.J., Peters L.C.J.M., Verbeek R.G.F.A. (2024). Flexible large-area ultrasound arrays for medical applications made using embossed polymer structures. Nat. Commun..

[26] Dahl T., Ealo J.L., Bang H.J., Holm S., Khuri-Yakub P. (2014). Applications of airborne ultrasound in human–computer interaction. Ultrasonics.

[27] Tovar-Lopez F.J. (2023). Recent Progress in Micro- and Nanotechnology-Enabled Sensors for Biomedical and Environmental Challenges. Sensors.

[28] Carotenuto R., Pezzimenti F., Della Corte F.G., Iero D., Merenda M. (2021). Ranging with Frequency Dependent Ultrasound Air Attenuation. Sensors.

[29] Turo A., Salazar J., Chavez J.A., Kichou H.B., Gomez T.E., De Espinosa F.M., Garcia-Hernandez M.J. (2003). Ultra-low noise front-end electronics for air-coupled ultrasonic non-destructive evaluation. NDT E Int..

[30] Mehta A., Vasudev H. (2024). Advances in welding sensing information processing and modelling technology: An overview. J. Adhes. Sci. Technol..

[31] Schmid S., Dürrmeier F., Grosse C. (2023). Spatial and Temporal Deep Learning in Air-Coupled Ultrasonic Testing for Enabling NDE 4.0. J. Nondestruct. Eval..

[32] Álvarez-Arenas T.G., Camacho J. (2019). Air-Coupled and Resonant Pulse-Echo Ultrasonic Technique. Sensors.

[33] Larsen O.N., Radford C., Slabbekoorn H., Dooling R.J., Popper A.N., Fay R.R. (2018). Acoustic Conditions Affecting Sound Communication in Air and Underwater. Effects of Anthropogenic Noise on Animals.

[34] Olisa S.C., Khan M.A., Starr A. (2021). Review of Current Guided Wave Ultrasonic Testing (GWUT) Limitations and Future Directions. Sensors.

[35] Chen H., Sun Q., Xuan L., Lin Z., Yang Z., Huang X., Li Z., Gao W., Ren J., Shi J. (2024). Ultrasonic technology for predicting beef thawing degree and endpoint. J. Food Eng..

[36] Orellana A.E.M., Mendler A., Schmid S., Grosse C.U. (2025). Predictive probability of detection curves for ultrasonic testing. NDT E Int..

[37] Dardouri A., Othmani C., Salah I.B., Zhang B., Njeh A. (2025). Guided waves in sandwich plates: Revealing an approximate threshold of contrast material properties for Legendre polynomial method limitations. Acta Mech. Sin..

[38] Kachanov V.K., Sokolov I.V., Karavaev M.A. (2025). Selecting optimum air gap length in air-coupled ultrasonic through-transmission testing of products made of polymer materials. Russ. J. Nondestruct. Test..

[39] Boccaccio M., Rachiglia P., Malfense Fierro G.P., Pio Pucillo G., Meo M. (2021). Deep-Subwavelength-Optimized Holey-Structured Metamaterial Lens for Nonlinear Air-Coupled Ultrasonic Imaging. Sensors.

[40] Jeger-Madiot N., Gateau J., Fink M., Ing R.K. (2017). Non-contact and through-clothing measurement of the heart rate using ultrasound vibrocardiography. Med. Eng. Phys..

[41] Jeng G.S., Chen S., Hsieh L.T., Lo M.T. (2025). Contactless Respiratory Waveform Estimation Using Ultrasound Planar Array. IEEE Open J. Ultrason. Ferroelectr. Freq. Control.

[42] Ambrosanio M., Franceschini S., Grassini G., Baselice F. (2020). A multi-channel ultrasound system for non-contact heart rate monitoring. IEEE Sens. J..

[43] Cailly W., Gonzalez-Diaz R., Nieminen H.J. (2018). Accuracy and robustness of an air-ultrasound method for non-contact heart rate and heart rate variability measurements Open Access. J. Acoust. Soc. Am..

[44] Shahshahani A., Zilic Z., Bhadra S. (2020). An ultrasound-based biomedical system for continuous cardiopulmonary monitoring: A single sensor for multiple information. IEEE Trans. Biomed. Eng..

[45] Wang T., Zhang D., Wang L., Zheng Y., Gu T., Dorizzi B., Zhou X. (2019). Contactless respiration monitoring using ultrasound signal with off-the-shelf audio devices. IEEE Internet Things J..

[46] Min S.D., Kim J.K., Shin H.S., Yun Y.H., Lee C.K., Lee M. (2010). Noncontact respiration rate measurement system using an ultrasonic proximity sensor. IEEE Sens. J..

[47] Singh A., Rehman S.U., Yongchareon S., Chong P.H.J. (2020). Multi-resident non-contact vital sign monitoring using radar: A review. IEEE Sens. J..

[48] Rehouma H., Noumeir R., Essouri S., Jouvet P. (2020). Advancements in Methods and Camera-Based Sensors for the Quantification of Respiration. Sensors.

[49] Gatzoulis L., Iakovidis I. (2007). Wearable and portable eHealth systems. IEEE Eng. Med. Biol. Mag..

[50] Chen A., Rhoades R.D., Halton A.J., Booth J.C., Shi X., Bu X., Wu N., Chae J. (2022). Wireless Wearable Ultrasound Sensor to Characterize Respiratory Behavior. Methods Mol. Biol..

[51] Massaroni C., Nicoló A., Sacchetti M., Schena E. (2021). Contactless methods for measuring respiratory rate: A review. IEEE Sens. J..

[52] Selvaraju V., Spicher N., Wang J., Ganapathy N., Warnecke J.M., Leonhardt S., Swaminathan R., Deserno T.M. (2022). Continuous Monitoring of Vital Signs Using Cameras: A Systematic Review. Sensors.

[53] Moon H.H., Lee G.Y., Kim G.S., Ra G.L., Jeong J.S. (2025). Bidirectional non-contact ultrasound imaging using MHz-band air-coupled ultrasound transducer for skin assessment: A feasibility study. Ultrasonics.

[54] Kawai K., Ohara R., Sato S., Ishii T., Izumi S., Kawaguchi H. Contactless respiration measurement system using 25-kHz spatial ultrasound Doppler sensor. Proceedings of the 2023 IEEE International Ultrasonics Symposium.

[55] Melo D.F., Silva B.M., Pombo N., Xu L. (2021). Internet of things assisted monitoring using ultrasound-based gesture recognition contactless system. IEEE Access.

[56] Sgambato B.G., Hasbani M.H., Barsakcioglu D.Y., Ibáñez J., Jakob A., Fournelle M., Tang M.T., Farina D. (2024). High performance wearable ultrasound as a human-machine interface for wrist and hand kinematic tracking. IEEE Trans. Biomed. Eng..

[57] Kim K., Kim S., Choi A. (2022). Ultrasonic Sound Guide System with Eyeglass Device for the Visually Impaired. Sensors.

[58] Panazan C.-E., Dulf E.-H. (2024). Intelligent Cane for Assisting the Visually Impaired. Technologies.

[59] Mousse M.A. (2022). Visually Impaired People Monitoring in a Smart Home using Electronic White Cane. Int. J. Comput. Sci. Inf. Technol..

[60] Bai J., Lian S., Liu Z., Wang K., Liu D. (2017). Smart guiding glasses for visually impaired people in indoor environment. IEEE Trans. Consum. Electron..

[61] Franceschini S., Ambrosanio M., Autorino M.M., Baselice F. (2024). A Novel Ultrasound System for Contactless Quantitative Measurements of Finger Tapping: A Feasibility Study. 2024 IEEE International Conference on E-health Networking, Application & Services (HealthCom), Nara, Japan.

[62] Sohl-Dickstein J., Teng S., Gaub B.M., Rodgers C.C., Li C., DeWeese M.R., Harper N.S. (2015). A Device for Human Ultrasonic Echolocation. IEEE Trans. Biomed. Eng..

[63] He K. (2025). Ultrasound-based human machine interfaces for hand gesture recognition: A scoping review and future direction. IEEE Trans. Med. Robot. Bionics.

[64] Huang Y., Yang X., Li Y., Zhou D., He K., Liu H. (2018). Ultrasound-based sensing models for finger motion classification. IEEE J. Biomed. Health Inform..

[65] Blum F., Jarzynski J., Jacobs L.J. (2005). A focused two-dimensional air-coupled ultrasonic array for non-contact generation. NDT E Int..

[66] Ma H., Wang Z., Cheng Z., He G., Feng T., Zuo C., Qiu H. (2022). Multiscale confocal photoacoustic dermoscopy to evaluate skin health. Quant. Imaging Med. Surg..

[67] Salim M.S., Abd Malek M.F., Heng R.B.W., Juni K.M., Sabri N. (2012). Capacitive micromachined ultrasonic transducers: Technology and application. J. Med. Ultrasound.

[68] Ji Y., Fan M., Li B., Gao G., Jiang Z. (2025). Rapid In Situ Coating of Covered Stents with Highly Tough, Biocompatible Membrane for Emergency Coronary Artery Perforation. Biomolecules.

[69] Fritze H. (2010). High-temperature bulk acoustic wave sensors. Meas. Sci. Technol..

[70] Matko V., Milanovič M. (2020). Detection Principles of Temperature Compensated Oscillators with Reactance Influence on Piezoelectric Resonator. Sensors.

[71] Carr A.R., Chan Y.J., Reuel N.F. (2023). Contact-Free, Passive, electromagnetic resonant sensors for enclosed biomedical applications: A perspective on opportunities and challenges. ACS Sens..

[72] Bera T.K. (2014). Bioelectrical impedance methods for noninvasive health monitoring: A review. J. Med. Eng..

[73] Seeber B.U., Kerber S., Hafter E.R. (2010). A system to simulate and reproduce audio–visual environments for spatial hearing research. Hear. Res..

[74] Zeqiri B., Scholl W., Robinson S.P. (2010). Measurement and testing of the acoustic properties of materials: A review. Metrologia.

[75] Chen J., Zhao J., Lin L., Sun X. (2019). Truncated Conical PVDF Film Transducer for Air Ultrasound. IEEE Sens. J..

[76] Turner R.C., Fuierer P.A., Newnham R.E., Shrout T.R. (1994). Materials for high temperature acoustic and vibration sensors: A review. Appl. Acoust..

[77] Drinkwater B.W., Wilcox P.D. (2006). Ultrasonic arrays for non-destructive evaluation: A review. NDT E Int..

[78] Angiulli G., Versaci M., Burrascano P., Laganá F. (2025). A Data-Driven Gaussian Process Regression Model for Concrete Complex Dielectric Permittivity Characterization. Sensors.

[79] Pratticò D., Laganà F. (2025). Infrared Thermographic Signal Analysis of Bioactive Edible Oils Using CNNs for Quality Assessment. Signals.

[80] Wang Z., Li S., Shen G. (2025). Advanced Sensory Hardware for Intelligent Eye-Machine Interfacing: From Wearables to Bionics. Adv. Funct. Mater..

[81] Al-Sakaji B.A.K., Al-Asheh S., Maraqa M.A. (2022). A Review on the Development of an Integer System Coupling Forward Osmosis Membrane and Ultrasound Waves for Water Desalination Processes. Polymers.

[82] He B., Ji X., Li G., Cheng B. (2024). Key technologies and applications of UAVs in underground space: A review. IEEE Trans. Cogn. Commun. Netw..

[83] Rathod V.T. (2019). A Review of Electric Impedance Matching Techniques for Piezoelectric Sensors, Actuators and Transducers. Electronics.

[84] Banafaa M., Muqaibel A.H. (2025). Tropospheric Ducting: A Comprehensive Review and Machine Learning based Classification Advancements. IEEE Access.

[85] Gao R., Liang M., Dong H., Luo X., Suganthan P.N. (2025). Underwater acoustic signal denoising algorithms: A survey of the state-of-the-art. IEEE Trans. Instrum. Meas..

[86] Liu M., Liu H., He X., Jin S., Chen P., Xu M. (2025). Research advances on non-line-of-sight imaging technology. J. Shanghai Jiaotong Univ. (Sci.).

[87] Pradhan S.S., Unnikrishnan L., Mohanty S., Nayak S.K. (2020). Thermally conducting polymer composites with EMI shielding: A review. J. Electron. Mater..

[88] Cai Y., Yu H., Cheng L., Guo S., Liu T., Chen D., Huang S., Hu Z., Wang Y., Zhou Y. (2023). Structure design, surface modification, and application of CNT microwave-absorbing composites. Adv. Sustain. Syst..

[89] Fekiač J.J., Krbata M., Kohutiar M., Janík R., Kakošová L., Breznická A., Eckert M., Mikuš P. (2025). Comprehensive Review: Optimization of Epoxy Composites, Mechanical Properties, & Technological Trends. Polymers.

[90] Šutka A., Lapčinskis L., He D., Kim H., Berry J.D., Bai J., Knite M., Ellis A.V., Jeong C.K., Sherrell P.C. (2023). Engineering polymer interfaces: A review toward controlling triboelectric surface charge. Adv. Mater. Interfaces.

[91] Hiremath N., Kumar V., Motahari N., Shukla D. (2021). An Overview of Acoustic Impedance Measurement Techniques and Future Prospects. Metrology.

[92] Pillai G., Li S.S. (2021). Piezoelectric MEMS resonators: A review. IEEE Sens. J..

[93] Ma J., Hu J., Li Z., Nan C.W. (2011). Recent progress in multiferroic magnetoelectric composites: From bulk to thin films. Adv. Mater..

[94] Zhang J., Wang J., Zhong C., Zhang Y., Qiu Y., Qin L. (2024). Flexible Electronics: Advancements and Applications of Flexible Piezoelectric Composites in Modern Sensing Technologies. Micromachines.

[95] Nivedhitha D.M., Jeyanthi S. (2023). Polyvinylidene fluoride, an advanced futuristic smart polymer material: A comprehensive review. Polym. Adv. Technol..

[96] Fiorillo A.S., Pullano S.A., Critello C.D. (2020). Spiral-shaped biologically-inspired ultrasonic sensor. IEEE Trans. Ultrason. Ferroelectr. Freq. Control.

[97] Pullano S.A., Bianco M.G., Critello D.C., Menniti M., La Gatta A., Fiorillo A.S. (2020). A Recursive Algorithm for Indoor Positioning Using Pulse-Echo Ultrasonic Signals. Sensors.

[98] Müller R., Kuc R. (2007). Biosonar-inspired technology: Goals, challenges and insights. Bioinspiration Biomim..

[99] Roy S., Azad A.W., Baidya S., Alam M.K., Khan F. (2022). Powering solutions for biomedical sensors and implants inside the human body: A comprehensive review on energy harvesting units, energy storage, and wireless power transfer techniques. IEEE Trans. Power Electron..

[100] Wu T., You D., Gao H., Lian P., Ma W., Zhou X., Wang C., Luo J., Zhang H., Tan H. (2023). Research Status and Development Trend of Piezoelectric Accelerometer. Crystals.

[101] Bibbò L., Angiulli G., Laganà F., Pratticò D., Cotroneo F., La Foresta F., Versaci M. (2025). MEMS and IoT in HAR: Effective Monitoring for the Health of Older People. Appl. Sci..

[102] Zhu J., Liu X., Shi Q., He T., Sun Z., Guo X., Liu W., Sulaiman O.B., Dong B., Lee C. (2020). Development Trends and Perspectives of Future Sensors and MEMS/NEMS. Micromachines.

[103] Birjis Y., Swaminathan S., Nazemi H., Raj G.C.A., Munirathinam P., Abu-Libdeh A., Emadi A. (2022). Piezoelectric Micromachined Ultrasonic Transducers (PMUTs): Performance Metrics, Advancements, and Applications. Sensors.

[104] Wong S.J., Roy K., Lee C., Zhu Y. (2024). Thin-film piezoelectric micromachined ultrasound transducers in biomedical applications: A review. IEEE Trans. Ultrason. Ferroelectr. Freq. Control.

[105] Xu T., Zhao L., Jiang Z., Guo S., Li Z., Ping Y. (2020). Equivalent Circuit Model for a Large Array of Coupled Piezoelectric Micromachined Ultrasonic Transducers with High Emission Performance. IEEE Trans. Ultrason. Ferroelectr. Freq. Control.

[106] Li Z., Zhao L., Li J., Zhao Y., Xu T., Liu Z., Luo G., Zhang S., Hu K., Hoffman T. (2021). Nonlinear behavior analysis of electrostatically actuated multilayer anisotropic microplates with residual stress. Compos. Struct..

[107] Svilainis L., Dumbrava V. (2005). Design of a Low-Noise Preamplifier for Ultrasonic Transducers. Ultragarsas (Ultrasound).

[108] Choi H. (2024). Design of Preamplifier for Ultrasound Transducers. Sensors.

[109] Zamora I., Ledesma E., Uranga A., Barniol N. (2020). Miniaturized 0.13-µm CMOS Front-End Analog for AlN PMUT Arrays. Sensors.

[110] Olivieri R., Barile G., Stornelli V., Ciarrocchi D., Fonte M., Zompanti A., Ferri G. (2025). A novel current-mode EMG interface. International Workshop Advances in Sensors and Interfaces (IWASI), Manfredonia, Italy.

[111] Ozer E., Kacar F. (2022). Current-mode PID controller using second-generation voltage conveyor (VCII). J. Circuits Syst. Comput..

[112] Olivieri R., Di Lizio G.A., Barile G., Stornelli V., Ferri G., Minaei S. (2025). Conveyor-based single-input triple-output second-order LP/BP and cascaded first-order HP filters. Electronics.

[113] Safari L., Barile G., Stornelli V., Ferri G. (2022). A Review on VCII Applications in Signal Conditioning for Sensors and Bioelectrical Signals: New Opportunities. Sensors.

[114] O’Reilly M.A., Hynynen K. (2010). A PVDF Receiver for Ultrasound Monitoring of Transcranial Focused Ultrasound Therapy. IEEE Trans. Biomed. Eng..

[115] Pullano S.A., Fiorillo A.S., Barile G., Stornelli V., Ferri G. (2021). A Second-Generation Voltage-Conveyor-Based Interface for Ultrasonic PVDF Sensors. Micromachines.

[116] Barile G., Pullano S.A., Fiorillo A.S., Ferri G. A Broadband Approach for the Generation and Reception of Low-Frequency Ultrasounds In-Air for Sonar Applications. Proceedings of the 2021 International Conference on e-Health and Bioengineering (EHB).

[117] Lin Y., O’Reilly M.A., Hynynen K. (2023). A PVDF Receiver for Acoustic Monitoring of Microbubble-Mediated Ultrasound Brain Therapy. Sensors.

[118] Rui G., Allahyarov E., Zhu Z., Huang Y., Wongwirat T., Zou Q., Taylor P.L., Zhu L. (2024). Challenges and opportunities in piezoelectric polymers: Effect of oriented amorphous fraction in ferroelectric semicrystalline polymers. Responsive Mater..

[119] Kashdan J.T., Shrimpton J.S., Whybrew A. (2003). Two-phase flow characterization by automated digital image analysis. Part 1: Fundamental principles and calibration of the technique. Part. Part. Syst. Charact..

[120] Boujenoui A., El Atlas N., Bybi A., Reskal H., Elmaimouni L. (2025). Advances in Crosstalk Reduction Techniques for Ultrasonic Transducer Arrays. Sensors.

[121] Weng C., Gu X., Jin H. (2024). Coded Excitation for Ultrasonic Testing: A Review. Sensors.

[122] Zhu B., Ma Y., Zhou Z., Guo W., Zhu J., Zhu X. (2025). Underwater Acoustic Signal Denoising with Diffusion-based Generative Models. Signal Process..

[123] Boris I., Barashok K., Choi Y., Choi Y., Aslam M., Lee J. (2025). Machine learning techniques in ultrasonics-based defect detection and material characterization: A comprehensive review. Adv. Mech. Eng..

[124] Yan J., Zhang Y., Jiao Z., Song L., Wang Z., Zhang Q., Liu Y., Qin W. (2025). Opportunities and challenges of ultrasonic diagnostic techniques for plant-based food monitoring: Principle, machine system, and application strategies. Crit. Rev. Food Sci. Nutr..

[125] Sethi A.K., Muddaloor P., Anvekar P., Agarwal J., Mohan A., Singh M., Gopalakrishnan K., Yadav A., Adhikari A., Damani D. (2023). Digital Pulmonology Practice with Phonopulmography Leveraging Artificial Intelligence: Future Perspectives Using Dual Microwave Acoustic Sensing and Imaging. Sensors.

[126] Schaller C., Penne J., Hornegger J. (2008). Time-of-flight sensor for respiratory motion gating. Med. Phys..

[127] Xu J., Gao X., Padasdao B.E., Boric-Lubecke O. Estimation of physiological sub-millimeter displacement with CW Doppler radar. Proceedings of the 2015 37th Annual International Conference of the IEEE Engineering in Medicine and Biology Society (EMBC).

[128] Zhu J., Zhang D., Shu Z., Samson P., Robinson C., Hao Y., Zhang T. (2025). Retrospective 4D-CT binning based on concurrent non-contact respiratory and cardiac phase tracking using millimeter wave (mmWave) radar. Med. Phys..

[129] Von Tscharner V. (2000). Intensity analysis in time-frequency space of surface myoelectric signals by wavelets of specified resolution. J. Electromyogr. Kinesiol..

[130] Alzahab N.A., Apollonio L., Di Iorio A., Alshalak M., Iarlori S., Ferracuti F., Monteriù A., Porcaro C. (2021). Hybrid Deep Learning (hDL)-Based Brain-Computer Interface (BCI) Systems: A Systematic Review. Brain Sci..

[131] Pullano S.A., Oliva G., Presta P., Carullo N., Musolino M., Andreucci M., Bolignano D., Fiorillo A.S., Coppolino G. (2025). A portable easy-to-use triboelectric sensor for arteriovenous fistula monitoring in dialysis patients. Sens. Int..

[132] Ramadas C., Janardhan Padiyar M., Balasubramaniam K., Joshi M., Krishnamurthy C.V. (2010). Delamination size detection using time of flight of anti-symmetric (Ao) and mode converted Ao mode of guided Lamb waves. J. Intell. Mater. Syst. Struct..

[133] Fan Z., Bai K., Chen C. (2024). Ultrasonic testing in the field of engineering joining. Int. J. Adv. Manuf. Technol..

[134] Ramaswami M. (2014). Network plasticity in adaptive filtering and behavioral habituation. Neuron.

[135] Mitra M., Gopalakrishnan S. (2016). Guided wave based structural health monitoring: A review. Smart Mater. Struct..

[136] Ostrysz M., Szczepaniak Z., Sondej T. (2026). Non-Contact Measurement of Human Vital Signs in Dynamic Conditions Using Microwave Techniques: A Review. Sensors.

[137] Guo X., Zhang Z., Ren Z., Li D., Xu C., Wang L., Liu W., Zhuge Y., Zhou G., Lee C. (2025). Advances in Intelligent Nano-Micro-Scale Sensors and Actuators: Moving toward Self-Sustained Edge AI Microsystems. Adv. Mater..

